# Impacts of COVID-19 on Owner's Veterinary Healthcare Seeking Behavior for Dogs With Chronic Conditions: An Exploratory Mixed-Methods Study With a Convenience Sample

**DOI:** 10.3389/fvets.2022.902219

**Published:** 2022-05-26

**Authors:** Sara C. Owczarczak-Garstecka, Tamzin Furtado, Taryn M. Graham, Imogen Lloyd, David A. Singleton, Lisa Wallis, Carri Westgarth

**Affiliations:** Department of Livestock and One Health, Faculty of Health and Life Sciences, Institute of Infection Veterinary and Ecological Sciences, University of Liverpool, Leahurst, United Kingdom

**Keywords:** dogs, chronic disease, COVID-19, delivery of veterinary healthcare, health literacy (HL), terminal care

## Abstract

This mixed-method study explored the impacts of the COVID-19 pandemic on owners' veterinary healthcare seeking, with particular focus on dogs with chronic conditions. A convenience sample of 719 UK dog owners completed an online survey (December 2020-January 2021). Differences in treatment provision and respondents' decisions to seek care across acute, preventative, chronic conditions and for end-of-life care were explored. Multivariable logistic regression models were used to identify factors associated with seeking care for any health issue compared to deciding against it, and urgency to seek care given symptom that could indicate chronic conditions. Open-ended questions were analyzed by thematic analysis. Significant (p-value < 0.05) differences in care seeking decisions were identified regarding access to veterinary care and the way treatment was provided across all health issues. The top reasons for not seeking care across all health issues were a lack of access to a veterinarian (30%, *n* = 56/187) and a reluctance for a dog to go to the clinic unaccompanied (20%, *n* = 38/187). Variables related to stronger dog-owner relationship, higher confidence in seeking care, perception of: benefits of veterinary care, dog's high susceptibility to illness and high severity of dog's condition, increased the odds of seeking, and urgency to seek, care. A dog's chronic illness diagnosis reduced the odds of seeking care during the pandemic, reportedly due to difficulties in accessing care for non-urgent issues. Qualitative analysis showed that limited access to routine consultations, delays in test results and restricted access to complementary treatments, led some owners of dogs with chronic conditions to believe that their dog's welfare had deteriorated during the pandemic. Pandemic control measures necessitated changes to how consultations were run. These changes were often viewed favorably, but dog-client separation during consultations were considered problematic, sometimes delaying veterinary advice-seeking, including for euthanasia. Separating owners from their dogs during veterinary consultations should be avoided wherever possible due to impacts on dogs, owners and healthcare seeking. Interventions to improve veterinary healthcare seeking could target attitudes toward benefits of seeking care, improve owners' self-efficacy and capitalize on the dog-owner bond. Such interventions should be implemented alongside interventions aimed at removing structural barriers to accessing healthcare.

## Introduction

Efforts to slow down the transmission of COVID-19 virus in the UK led to restrictions of individuals' movements, social interactions and work, including work of veterinary practices (1–5). During the first strict UK national lockdown introduced on the 23^rd^ March 2020, veterinary practices were only permitted to carry out emergency services (1, 6), consultations were run in a socially-distanced manner and wearing of personal protective equipment was common (7). The strict national restrictions were lifted on 13^th^ May 2020, however further national and regional lockdowns were in place throughout 2020–2021 and changes to veterinary practice protocols largely continued. The provision of veterinary healthcare was additionally disrupted as only veterinary professionals involved in food supply or provision of essential veterinary care were initially considered as critical workers and thus eligible to access in-person childcare or schools, enabling those with child-care responsibility to continue to work (8). In January 2021, the provision of essential veterinary care in England was no longer considered as a criterion for the crucial worker status (9), further disrupting the provision of veterinary care. Many dog owners acquired dogs during the pandemic, therefore further disruptions occurred at a time when the UK's dog population, and plausibly the demand for veterinary care, also increased (10, 11).

Veterinary clinics in the UK and globally adapted standard operating protocols to accommodate socially-distanced consultations (12). Although many dog owners worried about access to veterinary care for emergency and non-emergency health issues (12), nearly 97% (*n* = 1,794/1,843) of those who booked an emergency appointment and 100% (*n* = 40/40) who arrived at a veterinary clinic for an emergency without an appointment were able to access help (13). However, owners often struggled to book appointments, in particular for issues that were not life threatening and reported significant delays in accessing preventative healthcare [specifically vaccinations and neutering, (13)]. Additionally, changes to how consultations were run affected, and often tested or challenged, the veterinary-client relationship (12). Some dog owners welcomed the flexibility that came with using telemedicine and were content with their dog being examined without them being present (13), but most found this prospect deeply distressing (12). This, combined with a need to rely on remote consultations, led some owners to perceive the quality of care to be lower than pre-pandemic, which in turn led to delays in seeking care (13). Studies based in the USA showed that dog owners with disabilities and from underprivileged communities additionally struggled accessing veterinary healthcare due to difficulties in arranging transport and accessing relevant financing options (14, 15). Together, these challenges made work in the veterinary sector more stressful (16–18) and led to concerns regarding long-term public engagement with veterinary healthcare (6).

COVID-19-related measures also impacted on daily routines of many dog owners. Although specific guidelines regarding dog walking for England, Scotland, Wales and Northern Ireland differed, generally, during the lockdown period, members of the public were only permitted to leave the house for exercise purposes once a day, including for dog walking purposes (2–5). Owners walked their dogs for longer, but less frequently, they walked more locally and kept away from other dog walkers when out (19, 20). Most dog owners spent more time with their dog than before the pandemic and some also substituted walks and exercise outside of the house with exercise at home (19, 21). Many dog owners reported that the company of their dog during the pandemic was important to their mental health, overall resilience and helped them to feel less lonely (19–25).

Many dogs suffer from chronic health issues i.e. health conditions that prevail over a course of one year (26, 27). These animals often depend on regular access to veterinary healthcare and other healthcare services [e.g. physiotherapy, massage therapy; (28)], which were not available or restricted during the pandemic. For many musculoskeletal chronic conditions, such as arthritis, frequent short walks are also advisable (29), meaning that the pandemic may have also affected the routine management of such dogs.

The Health Belief Model is often used in human health research to understand how individual demographic and psychological characteristics, knowledge and beliefs about illness and treatment, previous health-seeking experiences and the design of the healthcare system shape health-seeking behavior (30, 31). Some of these factors have been identified in relation to dog owners' veterinary healthcare seeking. For example, owners' compliance with routine check-ups and adherence to a vaccination schedule is influenced by their education, normative beliefs (social norms shared with family/friends), bond with a dog and knowledge about the disease and vaccination (32, 33). Seeing dog vaccinations or routine check-ups as expensive or unnatural (33) and having difficulties with accessing veterinary services (34) has been identified as a barrier to seeking vaccinations. In addition, research suggests that dog owners do not prioritize treatment for chronic health issues, such as obesity and dental conditions, in the same way as veterinarians, potentially because they are not aware of signs of these conditions (35) and their negative long-term impact (26, 36, 37). It is plausible that dog owners may struggle to recognize signs of chronic pain in dogs [common with some chronic diseases, (38)], thus potentially delaying access to treatment for conditions like osteoarthritis (39). Understanding of associations between owner-, dog-, and veterinary-healthcare-design factors, and seeking veterinary care for chronic and other health issues in dogs, is still poorly understood and to date research into veterinary healthcare seeking has tended to focus primarily on routine check-ups and vaccinations.

Therefore, this study aimed to explore impacts of the COVID-19 pandemic on dog owners seeking veterinary healthcare in the UK, with particular attention paid toward owners caring for dogs with chronic health conditions. We hypothesized that changes in provision of veterinary healthcare during the pandemic were likely to have a more profound impact on dogs suffering with chronic health issues than those without such diagnoses. Specifically, our objectives were to:

1. Compare dog owners' experiences of seeking and accessing veterinary healthcare for chronic, emergency conditions and preventative healthcare during the pandemic;
2. Explore reasons for not seeking care during the pandemic;
3. Explore dog owners' experiences of caring for a range of chronic health problems before and during the pandemic and their future care-plans; and
4. Identify associations between owner-, dog-, and veterinary-healthcare-design factors and seeking veterinary care for chronic health conditions in dogs within the Health Belief Model framework.

## Methods

This study implemented a mixed- methods qualitative and statistical analyses approach to improve understanding and interpretation of findings by applying analytical triangulation (40).

### Participants

An anonymous online survey, promoted through social media, was open between 15^th^ December 2020 and 25^th^ January 2021. The study inclusion criteria were age over 18 years old; living in the UK and owning a dog at some point during the COVID-19 pandemic, defined here between 23^rd^ March 2020 (the first day of the first national lockdown in England) and 25^th^ January 2021 (when the survey closed).

### Materials

The questionnaire was comprised of nine sections summarized in Table 1 (see Appendix A for details). Both open- and closed-ended questions were used. Owners of multiple dogs were asked to answer the questionnaire thinking about the dog whose name starts with a letter that appears earlier in alphabet.

**Table 1:** Summary of themes of questions used in the survey.

**Questionnaire section**	**Subject of questions**
About your dog	Sex, age, neuter status, source and date of acquisition, breed, and size. Monash Dog-Owner Relationship Scale [MDORS, (41)], Inclusion of Other in the Self (42, 43).
About you	Age, gender, education, household, living arrangements (living alone or with others), current number of dogs and number of dogs owned as an adult.
About your vet	Reasons for selecting the current vet, duration of attendance at the current veterinary practice, number of visits before and since the pandemic (and number of visits specifically for a chronic issue), duration of a visit (including travel time)
About COVID-19 in your area	Respondent's and household members COVID-19 symptoms, self-declared vulnerability to COVID-19 and worries regarding income caused by COVID-19.
Veterinary care during the COVID-19 pandemic: acute care/ standard preventative care/end-of-life-care/ chronic health conditions	In each section, the respondents were asked about reasons for potentially seeking treatment, whether they decided to seek treatment and if so, when and if they manage to access it, how care was received (e.g., a “dog was handed over to the vet/ vet nurse” or “owner was able to enter the practice with a dog”). Respondents who stated that they chose not to seek care/ were unable to access it were asked why this was the case. An open-ended question was included asking all respondents about factors taken into consideration when seeking care on this occasion.
Caring for dog's chronic illness^*^	Respondents who confirmed that their dog has a chronic condition were asked when the dog was diagnosed (before or since the pandemic), whether the treatment was covered by insurance, how the treatment/ medications prescribed by the veterinarian affect dog's behavior, if additional treatments (not prescribed by the veterinarian) were tried, and if so, how did they affect dog's behavior. These respondents were also asked to described how, if at all, caring for their dog was affected by COVID-19.
Knowledge and General attitudes to veterinary healthcare	Attitudes toward healthcare, e.g. “I care about my vet's views about how I manage my dog's health”, “My vet thinks that providing my dog with regular check-ups or treatment is important”: answers were presented on a 5-point Likert scale with strongly agree/ strongly disagree used as anchors.
	“The treatment provided by my veterinarian is necessary to manage my dog's health” and “Interrupting the treatment would be very risky”^*^
Urgency to seek care	Questions presented symptoms that could indicate chronic health conditions and asked respondents how quickly they would seek veterinary care, (e.g., Please indicate how long you would wait to contact your veterinarian in the following circumstances: If your dog became lame without having any visible injury or accident or If you noticed your dog bumping into objects). Answers were presented on a 5-point Likert-scale with options: Immediately seek an emergency appointment; On the same day to seek an appointment as soon as possible; Within a week, if the condition didn't improve; Within a month, if the condition didn't improve; I would not contact the vet for this.
Managing your dog's health in the future	Open-ended free-text question about owners' future plans for management of dog's health

* *Asked only if the respondent confirmed that a dog has a chronic health issue*.

### Data Handling

Data were de-duplicated by removing (*n* = 15) the same occasions of seeking care described multiple times (e.g., as an answer to questions regarding seeking care for acute health issues, chronic health issues and preventative care). Data cleaning included re-coding responses described in the free-text boxes as “other” into pre-existing categories where possible. Variables with multiple response options (e.g., household income, education) were pooled into 2–3 options ahead of multivariable logistic regression analysis (see Appendix A). A binary variable “Covid-19 experience” (yes/ no) was created by combining responses to questions “Have you experienced suspected COVID-19 disease symptoms” and “Has anyone else in your household experienced suspected COVID-19 symptoms” so that a positive response to either of these questions was recorded as a Yes. A binary variable “Sought help for any health issues” (yes/no) was created by pooling responses to questions “Did you seek veterinary advice, care or treatment since the beginning of the restrictions imposed due to the COVID-19 pandemic” asked about acute/ chronic conditions/ preventative healthcare/ end-of-life treatments. Response “Yes, I sought to access veterinary advice, care or treatment” to any of the health conditions was coded as “yes” and otherwise a response of “Yes, I considered it, but at that time I decided against accessing veterinary advice, care or treatment” or “No, I did not consider seeking veterinary advice care or treatment at the time” was coded as “no”. Surveys (*n* = 9) where respondents stated that they did not potentially need to seek help for any conditions were removed from the analysis. A binary variable (“urgent”/ “not urgent”) was created by dividing the combined score on questions about urgency to seek care as below or above the mean.

As the Monash Dog Owner Relationship Scale (MDORS) questionnaire consists of three sub-scales: Dog-Owner Interaction (9 questions, hereafter MDORS interactions sub-scale), Emotional Closeness (10 questions, hereafter MDORS closeness sub-scale) and Perceived Cost (9 questions, hereafter MDORS cost sub-scale), a total score as well as score for each sub-scale was calculated in accordance with published instructions (41). For questions that were a part of MDORS questionnaire or aimed to assess the urgency to seek care, single missing responses were replaced with a median for that sub-scale (MDORS) or across all responses (urgency to seek care). Where more than one response was missing, data were excluded from the analysis.

**Figure 1:**
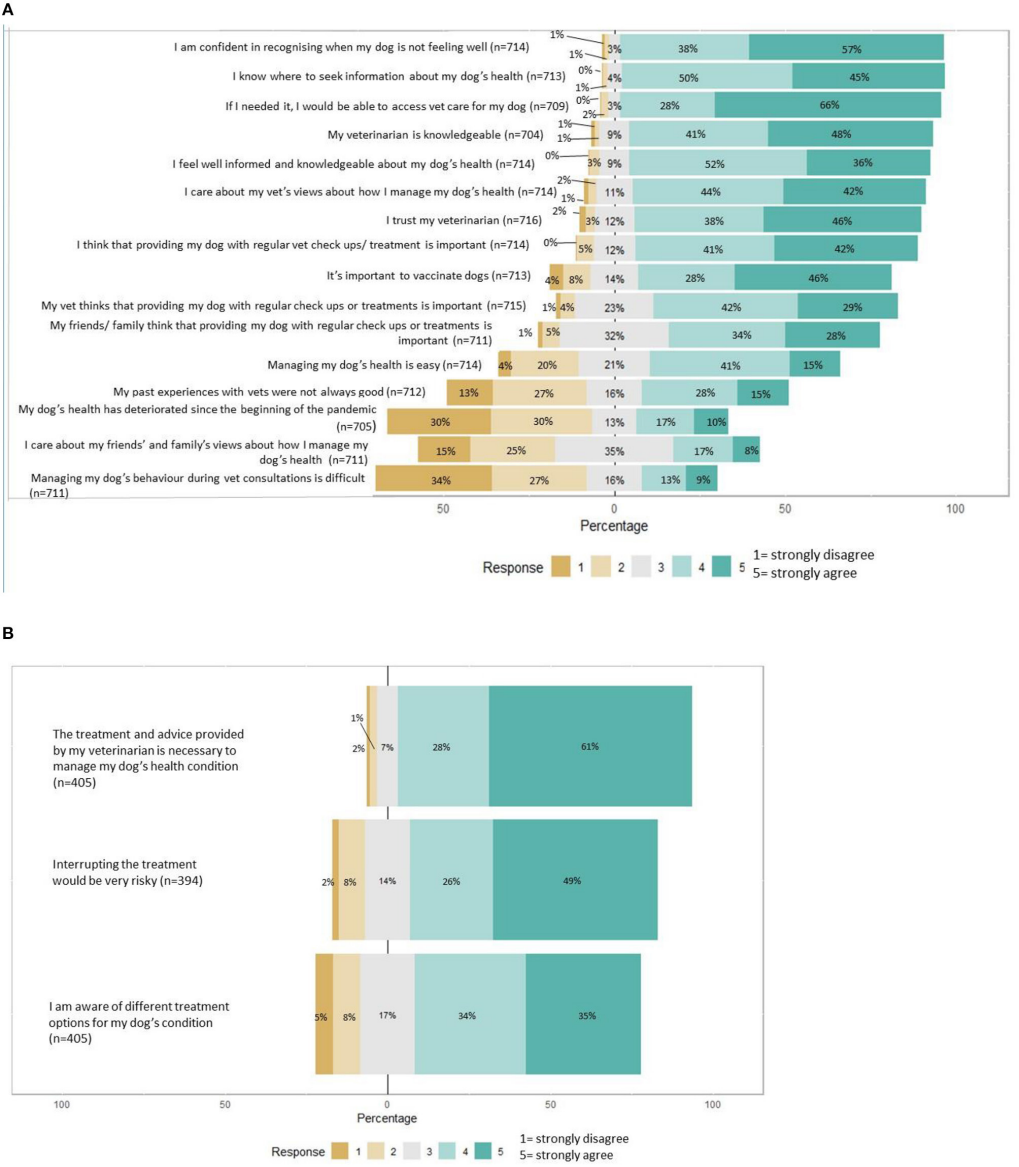
Summary of attitudes to seeking dog healthcare. **(A)** Summary of dog health-related attitudes among the whole population. **(B)** Summary of further dog health-related attitudes for owners of dogs with chronic helath issues.

Following the approach taken by Beyene et al. (34), questions assessing knowledge and general attitudes to veterinary healthcare (summarized later in Figure 1) were mapped onto constructs of the Health Belief Model (HBM). Constructs considered here included:

- Perceived susceptibility, i.e., belief about developing or contracting a condition;
- Perceived severity, i.e., belief regarding how serious the condition is or the perceived risk associated with a condition being untreated;
- Perceived benefits, i.e., beliefs regarding benefits of actions likely to reduce the threat of illness or contracting it, including benefits nor directly related to health (e.g., complying with social norms, financial gains, being perceived as responsible;
- Perceived barriers, i.e., negative aspects of taking health-related actions; and
- Self-efficacy, i.e., confidence that one's actions will lead to the desired outcome (44).

The sixth potential facet, “cues to action,” was not included as cues are often unconscious and therefore difficult to study via a survey (45).

### Quantitative Data Analyses

The demographic variables were summarized with descriptive statistics. Data distribution was checked visually and with Shapiro-Wilk tests to decide on suitability of parametric or non-parametric tests. Chi-square tests with Bonferroni corrections for multiple comparisons were used to compare the mode of treatment delivery (e.g., delivered as normal compared to dog examined without the owner) and respondents' ability to access healthcare for different health issues (acute/ preventative/ chronic/ end-of-life treatment). The mean monthly number of vet visits before and during the pandemic for dogs with/without chronic conditions was compared with paired Wilcoxon signed ranked tests.

Internal reliability of MDORS sub-scales, questions used to assess urgency to seek care and HMB construct was explored with Cronbach alpha (see Appendix B for how questions were mapped on the HBM constructs and for the detailed results of the reliability analysis).

Questions “It's important to vaccinate dogs” and “I think that providing my dog with regular veterinary check-ups or treatment is important” made the Perceived susceptibility HBM construct; questions “The treatment provided by my veterinarian is necessary to manage my dog's health” “Interrupting the treatment would be very risky” made the Perceived severity construct; “The treatment and advice provided by veterinarian is necessary,” “I trust my veterinarian,” “My veterinarian is knowledgeable,” “I care about my vet's views about how I manage my dog's health,” “My vet thinks that providing my dog with regular check-ups or treatment is important,” “My friends and family think that providing my dog with regular check-ups is important” made the Perceived benefits construct. Finally, “Managing my dog's health is easy,” “I feel well informed and knowledgeable about my dog's health,” “I know where to seek information about my dog's health,” “I am confident in recognizing when my dog is not feeling well,” “I am aware of different treatment options for my dog's condition,” “If I needed to, I would be able to access veterinary care for my dog” made the Self-efficacy construct. Other questions were fitted within logistic regression models individually (see Appendix B for reliability analysis).

Three logistic regression models were constructed, with the following outcome variables: (1) seeking care for any health issues; (2) urgency to seek care (analysis of the entire dataset), and (3) urgency to seek care (analysis of the subset of dogs with a chronic condition, as respondents who confirmed a chronic condition diagnosis were asked additional questions that could be included in modeling). Predictive variables included in all three models were: dog and owner demographic variables, dog-owner relationship (MDORS), household income and concerns about loss of income due to the pandemic, mean monthly number of vet visits before and since the pandemic, length of attendance at the veterinary practice, owners'/ household experiencing COVID-19 symptoms and owner's vulnerability to COVID-19. Depending on reliability level, Knowledge and General attitudes to veterinary healthcare questions (total score per construct/ statement) were included within all the logistic regression models as HBM constructs (α > 0.7) or as individual statements (α < 0.6). Constructs that yielded an α = 0.6–07 were tried within the models as individual statements and as constructs and the version that led to a better model prediction (see below) was selected. Therefore, HBM constructs (Perceived susceptibility, benefits, and self-efficacy) and individual statements corresponding to the barriers construct were included in all three models. Reasons for seeking care (acute/ preventative/chronic/ end-of-life care) were included in model 1 or 2. The first model also included a total score on urgency to seek care questions. Perceived severity construct and information about dog's insurance were included in the third model for the subset of dogs with chronic health condition diagnosis (questions about severity/ insurance were only asked if the respondent confirmed that a dog has been diagnosed with a chronic condition).

Correlation matrices of predictive variables were constructed first to avoid using correlated items (i.e., r > 0.7). All models were built as generalized linear models (GLMs) by backward elimination, starting with all predictive variables. General Additive Models were used to determine if polynomial functions for continuous variables provided a better fit. Comparisons between models with a linear, quadratic or cubic function were carried out using ANOVA Likelihood Ratio Test. Interactions between predictive variables were assessed with *post-hoc* pairwise comparisons with Tukey *p*-value corrections for multiple comparisons. The final variables left in the models were determined by significant *p*-values (<0.05) and using ANOVA Chi-Square analysis to identify if all remaining variables were significantly reducing the residual deviance. Models' predictive power was assessed with McFadden's pseudo R^2^ and Receiver Operating Characteristic (ROC) curves measured with c-statistic. Goodness of fit was assessed with Hosmer-Lemenshow (H-L) statistic. All analyses were conducted in R (46).

### Qualitative Data Analysis

Semantic inductive thematic analysis on open-ended questions was carried out. Inductive coding means that codes were assigned to summarize the data rather than to reflect an existing theory or pre-defined categories (47). Semantic coding (i.e., coding driven by the explicit content of the data) was deemed most suitable due to the often brief nature of free-text responses. The analysis followed the process outlined by Braun and Clarke (48). Briefly, after familiarization with the text, responses were coded line-by-line by two co-authors (SCOG and IL). Codes, aimed to summarize and condense the meaning expressed within each line (49), were assigned iteratively and updated as coding progressed, so that the coding scheme was continuously revised. The revised coding scheme was applied to the whole dataset and coding discrepancies were removed following a discussion between the co-authors. Codes were compared and similar codes were grouped to develop domain summaries, i.e., groupings of related codes (50). The final themes were created by comparing the relationships between codes within each domain summary and between the domains (48, 49). Direct quotes are used to illustrate themes. All coding was carried out in NVivo [v.2, QSR, (51)].

## Results

The survey was started by 1034 respondents, of whom 726 (70%) submitted a finished survey. Six respondents were excluded due to not meeting inclusion criteria, therefore 720 responses were used in the analysis. Below we describe the demographic data, COVID-19 variables, owner-vet relationship, health attitudes and pattern of responses to urgency to seek care questions. We then summarize owner's care routine for dogs with chronic health issues before the pandemic, before presenting analysis of owner's interactions with veterinary healthcare during the pandemic, reasons for not seeking care, predictors of seeking care and urgency to seek care and future plans regarding engagement with healthcare.

### Dog and Owner Characteristics

Based on the Inclusion of Self in others scale (42, 43), the majority (*n* = 481, 67%) of respondents had a strong relationship with their dog; 27% (*n* = 105) had a moderate relationship and 6% (*n* = 39) had a weak relationship. The mean total score on the MDORS scale was 117.1 (median =118) and the mean and median for the MDORS closeness, cost, and interactions sub-scales was 44.0 (median = 44), 38.2 (median = 39) and 35.6 (median = 36), respectively. The closeness and cost sub-scales had an excellent internal reliability (α > 0.8) and the interactions sub-scale had a reliability of α = 0.48 (95% CI 0.4–0.52). However, as the questionnaire has been previously validated (41), all sub-scales were included in the analysis in their entirety. The Cronbach alpha for Inclusion of Self in Others scale was poor (α = 0.23, 95% CI 0.18–0.25), therefore this variable was dropped from the analysis. Further dog and owner characteristics are summarized in Table 2.

**Table 2:** Dog and owner characteristics.

**Dog characteristics (** ***n*** **, %)**	
Sex	Male (*n* = 359, 50) Female (*n* = 350, 50) Missing information (*n* = 2, 0.1)
Age	Mean age: 82.0 months (6.8 years); SD = 52.2 months Median 72 months (6 years); IQR = 84 months
Neuter status	Neutered (*n* = 550, 77) Unneutered (*n* = 168, 23) Unknown (*n* = 1, 0.1)
Most common breeds	Cross-breed/ mongrel (*n* = 93, 13) Labrador Retriever (*n* = 66, 9) Border Collie (*n* = 45, 6) Miniature Schnauzer (*n* = 33, 5) Cocker Spaniel (*n* = 30, 4)
Size	Toy (*n* = 29, 11) Small (*n* = 171, 24) Medium (*n* = 282, 39) Large (*n* = 227, 32) Giant (*n* = 11, 2)
Timing of acquisition	Acquired before the pandemic (n=646, 90) Acquired during the pandemic (n=72, 10)
Source of acquisition	Commercial or hobby breeder (*n* = 403; 56) Dog shelter/ rescue (*n* = 213; 30) Other source^**^ (*n* = 104; 14)
Number of dogs in the household	One (*n* = 380, 53) Two (*n* = 182, 25) Three or more (*n* = 120, 17) Dog passed away during the pandemic (*n* = 34, 5)
**Owner characteristics (** ***n*** **, %)**	
Gender	Woman (*n* = 665, 93); Man (*n* = 43, 6) Prefer not to say (*n* = 7, 1) Non-binary (*n* = 2, 0.3)
Age	<50 years of age (*n* = 418, 58) >50 years of age (*n* = 292; 41) Prefer not to say (*n* = 6, 1)
Education	Educated to a degree level or above (*n* = 407, 57) Educate below a degree level (*n* = 303, 43)
Living arrangements	Living with others (*n* = 582, 82) Living alone (*n* =129, 18)
Dog-ownership experience	First time owning a dog as an adult (*n* = 277, 39) Owned previous dogs during adulthood (*n* = 436; 61)
Household income	Within or above UK's median (*n* = 473, 66) Below UK's median (*n* = 92, 13) Prefer not to say (*n* = 145, 20)
Concerns regarding the impact of the pandemic on financial security	Unconcerned (*n* = 314, 44) Neither concerned nor unconcerned (*n* = 80, 11) Concerned (*n* = 275, 38) Prefer not to say (*n* = 50, 7)

^*^The UK median in 2021 was £29,900 annually (52).

** *Other sources included: guide dog organizations, farms, friends/ family*.

### COVID-19 Related Variables

At the time of survey completion (15^th^ December 2020- 25^th^ January 2021), most (84%, *n* = 598) respondents had not experienced any COVID-19 symptoms, though 17% (*n* = 118) had. Among those who lived with others, most reported that no one else in their household had experienced COVID-19 symptoms (76%, *n* = 542), whereas 12% (*n* = 89) reported that other household members had experienced COVID-19 symptoms. Most respondents (*n* = 86%, *n* = 616) were not officially classified as vulnerable to COVID-19 (i.e. they have not received an official government notification letter). The remaining 6% (*n* = 46) were formally notified that they were particularly vulnerable and should be shielding, and 8% (*n* = 55) considered themselves vulnerable, although they did not receive the formal letter.

### Owner's Attitudes to and Relationship With the Vet and Urgency to Seek Care

Most respondents had been clients at their current veterinary practice for over 7 years (*n* = 253, 35%), 15% (*n* = 108) for <1 year; 11% (*n* = 80) for 3–5 years; 10% (*n* = 74) for 5–7 years and 0.3% (*n* = 2) could not remember. Most respondents (34%; *n* = 240) chose their vet practice because someone recommended it to them. Other reasons included: nearby location (*n* = 190; 27%), affordable prices (*n* = 68; 10%), friends or family attending the same practice (*n* = 64; 9%), vets taking time to explain things clearly (*n* = 61; 9%), access to specialist services (n=28; 4%), practice or services offered by it being covered by the insurance (*n* = 17; 2%) and other reasons (*n* = 47, 7%).

Questions that summarize owner's attitudes to healthcare are shown in Figure 1. Overall, owners in this sample were confident about seeking care and recognizing signs of poor health in their dog; responses to these questions were homogenous. Owners also agreed with the importance of preventative healthcare. There was a much greater diversity of responses, indicative of differences in experience, to questions about ease of managing dog's health, past experiences with veterinary healthcare, dog's health deteriorating during the pandemic and management of dog behavior during veterinary consultations.

**Figure 2:**
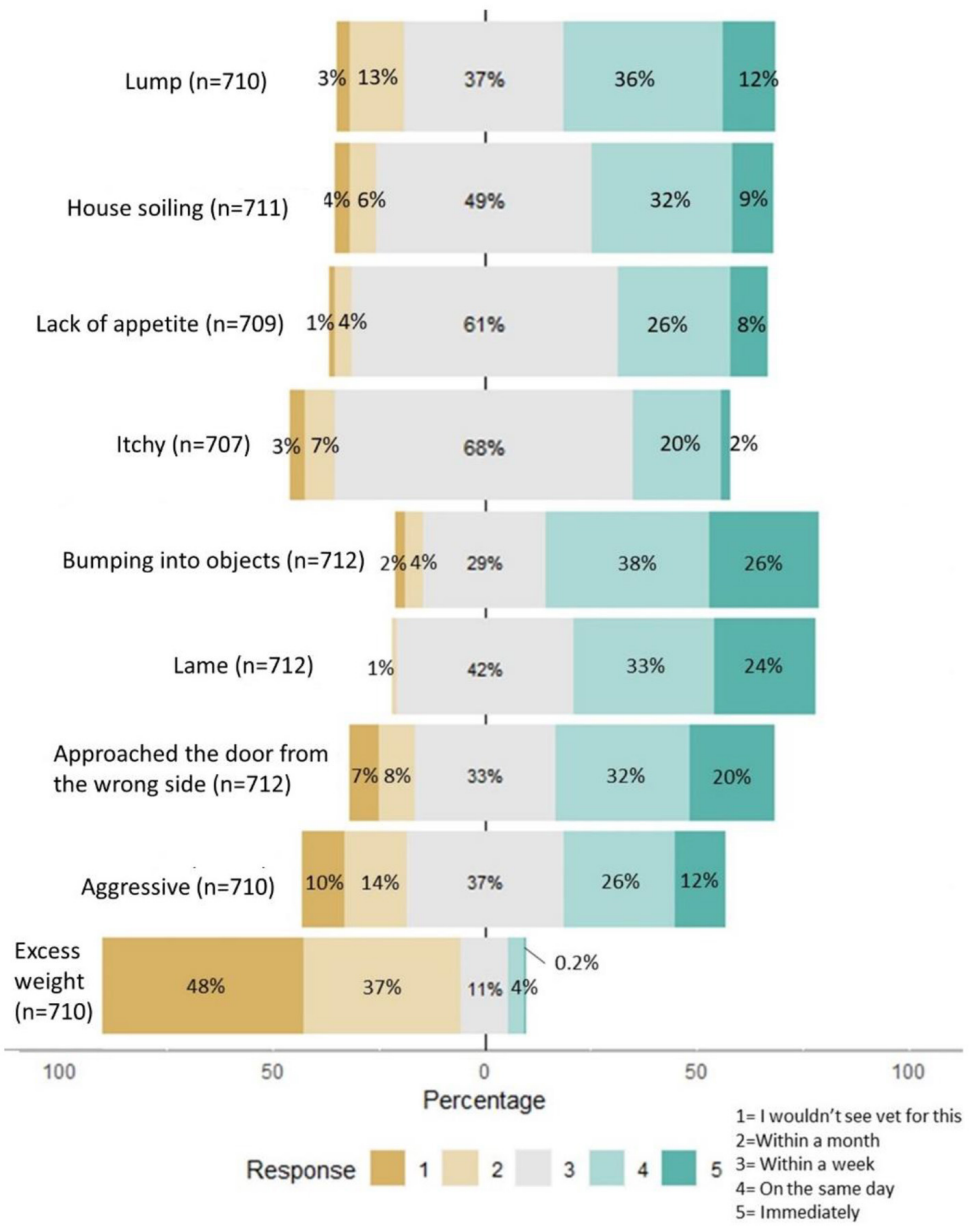
Summary of responses to questions about urgency to seek care given dog showing symptoms that could indicate common chronic health conditions.

Internal reliability of questions about urgency to seek care was excellent (α = 0.8, 95 CI% = 0.78–082). Therefore, the total score for these questions was treated as a single construct of urgency to seek care. Of all conditions listed in the hypothetical scenarios, owners reported the least urgency to seek veterinary healthcare if their dog was over-weight or aggressive (Figure 2). The greatest urgency to seek care was reported for dog becoming lame or bumping into objects.

### Management of Dog's Health Pre-pandemic (Dogs With Chronic Health Issues)

Owners who reported that their dog had chronic health problems were asked about their normal (i.e., pre-pandemic) health care management routine. Qualitative analysis identified three themes that summarize healthcare management: “Monitoring dog's health,” “Adapting care routines and owner's lifestyle” and “Financial commitment.” The first two themes were brought up by the majority of respondents. Although the third theme was not frequently discussed, it is important to consider it and document this experience, as financial commitment may be proportionally greater among dog owners who are not as affluent as those taking part in this study.

#### Monitoring Dog's Health

In addition to seeking preventative healthcare, annual wellness checks or treatment for acute health issues, owners of dogs with chronic health conditions also commonly sought veterinary care to monitor the dog's health and deliver specialist treatment or therapy:

“*He has to have blood/ urine tests every 6 months due to the medication he is on (…) Weekly hydrotherapy sessions planned - not only good for his conditions, but as regular therapist they can spot issues that I may have missed.”* (Respondent 11)

#### Adapting Care Routines and Owner's Lifestyle

Lifestyle adaptations that some dog owners made to manage their dog's health were common and sometimes substantial:

“*Every 2 hrs day and night I was taking him out and he'd sleep on my bed with me as he was having seizures and I didn't want him on his own. I didn't leave him alone for the last few months of his life as I know it was coming and didn't want him to pass away on his own”*. (Respondent 571)

Other reported adaptations and care given are summarized in Table 3.

**Table 3:** Management tools/ methods for managing health of dogs with chronic health conditions.

**Method**	***n* (%)**
No other ways	118 (13)
Weight management	86 (9)
Home adaptations, e.g., Installing ramps, non-slip carpets	143 (15)
Modified exercise regime, e.g., frequent but short walks, regular exercise	141 (15)
Nutrition, e.g., raw feeding; specific dietary formula, exclusion diet, soaking food to aide chewing, supplements believed to aid arthritis, prescribed medications	169 (18.)
Homeopathy	48 (5)
Herbal, e.g., over the counter and home-made herbal remedies	43 (5)
Acupuncture	20 (2)
Magnetic field therapy	10 (1)
Laser therapy	37 (4)
Hydrotherapy	70 (7)
Physiotherapy, e.g., with a trained practitioner; guided physiotherapy at home (e.g., over Zoom)	58 (6)
Massage	4 (0.4)
Other^*^	35 (4)

* *Other approaches included: behavior modification (n = 2, 0.2%), shampoo and other skin care products (n = 2, 0.1%); chiropractic therapy, wearing a coat, Galen myotherapy, using heat mats, immunotherapy, red light therapy, Reiki and stem cell therapy (n = 1 each)*.

#### Financial Commitment

Some owners described substantial financial commitment linked with caring for a dog with chronic issues:

“*When we had [our dog] euthanised and I went to pay her final bill I asked how much we had spent at that practice it came to £56,000 this did not include a couple of stays at an emergency vet hospital and cataract surgery at an optical vet, or ongoing supplies sourced online. I would guess that would increase the total to nearer 75k in total. Wouldn't change it for the world though and would have spent double that to keep her going, unfortunately money couldn't buy that.”* (Respondent 249)

### Interactions With Veterinary Healthcare Services During the Pandemic

#### Quantitative Findings

The five most common acute health issues that respondents potentially needed treatment for were: gastroenteritis (*n* = 72, 13%), joint/ligaments problems (*n* = 61, 11%), skin infection or other skin issues (*n* =47, 8%), ear infection (*n* = 44, 8%) and seizures (*n* = 32, 6%). The five most common preventative issues were: vaccination (primary or booster, *n* = 284, 58%), deworming treatment (*n* = 72, 15%), flea treatment (*n* = 60, 12%), neutering (*n* = 14, 3%) and nail trimming (*n* = 12, 2%). The corresponding chronic health issues were: osteoarthritis and other orthopedic conditions (*n* = 128, 41%), epilepsy (*n* = 38, 13%), endocrine disorder, allergies (*n* = 24, 7% each) and skin problems (*n* = 23, 7%). Finally, the most common reasons for potentially needing end-of-life care were: cancer (*n* = 38, 31%), age-related poor health (*n* = 29, 24%), heart failure (*n* = 11, 8%), epilepsy (*n* = 7, 6%) and kidney disease, osteoarthritis (*n* = 5, 4% each).

There was no difference in the monthly number of veterinary visits for dogs with chronic issues when comparing before and since the pandemic (pre-pandemic and during-pandemic median number of visits 0.30 and 0.33, respectively, *p* = 0.8). Dogs without chronic health conditions reported significantly more veterinary visits since the pandemic began than before (pre-pandemic and during-pandemic monthly median number of visits 0.2 and 0.3 respectively, *p* < 0.001).

Significant differences in healthcare seeking decisions between health conditions were identified (Table 4; X^2^ = 32.5, *p* < 0.001). Compared to acute health issues, those who potentially needed preventative treatment more often did not consider seeking it (*p* < 0.001) and did not manage to access it (*p* = 0.03). Those who potentially needed end-of-life care more often did not consider seeking it compared to those who needed care for acute issues (*p* = 0.01).

**Table 4:** Numbers (%) of respondents who potentially needed, considered, sought, accessed and did not manage to access veterinary care for acute, preventative, chronic health issues and end-of-life-care for their dogs during the COVID-19 pandemic.

**Type of issue**	**Acute**	**Preventative**	**Chronic**	**End of life**
Potentially needed treatment (*n*)	549	479	354	119 (116 continued with this section)
Did not consider seeking care (%)	21 (4)	54 (11)	23 (7)	13 (11)
Considered seeking care, but decided against it (%)	17 (3)	28 (6)	21 (6)	9 (8)
Sought care (%)	508 (93)	394 (82)	310 (88)	94 (81)
Accessed care (%)	489 (89)	359 (75)	295 (83)	86 (74)
Did not manage to access care (%)	18 (3)	34 (7)	15 (4)	7 (6)

There were significant differences in how treatment was delivered between health issues (Table 5; X^2^ = 167.6, *p* < 0.001). Compared to acute health issues and preventative treatments, seeking end-of-life care more often involved treatment as usual (including with small precautions i.e., wearing a mask/ using a hand sanitiser and maintaining social distance within the clinic; *p* < 0.001 for both acute and preventative healthcare). Compared to acute health issues and preventative treatments, seeking end-of-life care was more often carried out outdoors, e.g., in a carpark or practice garden (*p* < 0.001 for both acute and preventative healthcare). Compared to acute health issues and preventative treatments, seeking end-of-life care less often involved handing a dog over for the consultation (*p* < 0.001 for both acute and preventative healthcare). A similar pattern was observed for a comparison between end-of-life care and treatment for chronic health issues; end-of-life care was significantly more often received as usual (*p* = 0.01), outdoor (*p* < 0.001) and less often involved owner handing a dog over (*p* < 0.001). No other significant pairwise comparisons of how treatment was received between health issues were identified. The other modes of accessing care were not compared due to a small sample size/ not being utilized for all health issues (see Table 5).

**Table 5:** Comparison of how care for acute, preventative, chronic health issues and end-of-life health issues was received during the COVID-19 pandemic (%, *n*).

**Type of issues/ how was care received (% or n)**	**Acute**	**Preventative**	**Chronic**	**End-of-life-care**
Treated as usual (%)	128 (15)	97 (20)	89 (21)	33 (28)
Treated outdoors (%)	68 (8)	41 (8)	40 (10)	23 (20)
Treated without the owner (%)	369 (44)	200 (40)	179 (43)	12 (10)
Telephone advice only (%)	91 (11)	24 (5)	37 (9)	0
Email/app advice only (%)	25 (3)	6 (1)	9 (2)	0
Telephone advice & called into practice (%)	68 (8)	21 (4)	19 (5)	0
Collected meds only (%)	84 (10)	96 (19)	44 (11)	0
Other (%)	10 (1)	13 (3)	6 (1)	22 (19)^*^
TOTAL (across all categories, n)	843	498	417	110

* *Of the 22 dogs, 6 were euthanised at home, 2 died before owner reached the vet practice and in the case of 9 owners decided not to euthanise at that point*.

### Qualitative Findings - Experiences of Seeking Veterinary Care During the Pandemic

Owners' experiences of seeking and accessing care were very varied, reflecting different ways in which veterinary practices adapted their work to be COVID-19 secure and possibly differences in restrictions dependent on the time of when care was sought. The main qualitative themes that reflect dog owners' experiences of accessing and using healthcare are: “Accessing appointments,” “Change in consultation settings,” “Experience of quality of care” and “Impact on owner's finance.” Overall, the first three themes were discussed commonly. Albeit the fourth theme was not brought up frequently, it is important to discuss it as impact on owner's finance may have been more prevalent in the general population than in our data.

#### Accessing Appointments

Approximately half of the respondents who commented on the subject of appointment accessibility said they had no difficulties accessing check-up appointments for chronic issues and even preventative care:

“*I feel confidant [sic] that I can contact my vet via phone if required or get an appointment for an acute or worsening chronic condition. The emergency service is still in operation which reassures me.”* (Respondent 107)

However, roughly half of respondents who shared their experiences of accessing appointment said that booking an appointment for preventative treatments or regular check-up (for chronic issues or an annual health check) was difficult or impossible:

“*Would really like to be able to have regular check-ups again, but I have no idea when that will be possible. Would be nice to feel that the vet was welcoming whatever the issue, but that's not the case at the moment. Very much feels like non-emergency issues are slipping through the gaps as vets are pushing the 'emergencies only' approach hard.”* (Respondent 606)

In addition, owners of dogs with chronic health issues often relied on complementary therapies or additional non-veterinary services which were also hard to access:

“*(…) I take him to hydrotherapy [for dog's arthritis] and manage it that way as it's not bad and he doesn't seem in pain from it as such. Every lockdown the pool has to close which is awful as the longer he misses his swims the more his muscle wastes away. We build it back up once we can go swimming again but it's stressful and a worry for me.”* (Respondent 405)

#### Change in Consultation Settings

The vast majority of respondents commented on at least small changes in consultations settings. Many described how their consultation was carried out remotely via telemedicine solution:

“*I consulted verbally [over the phone] and sent photos because it was not life-threatening. Prior to Covid I would have visited the vet.”*(Respondent 188)

However, the main change to owners' experiences of accessing care was that often, owners were unable to accompany a dog into the practice. When allowed in, owners were not always permitted to be close to their dog due to social distancing measures. For a small number of dog owners this was not a problem: they believed that their dog had a good relationship with veterinary staff and was happy to go into the clinic alone and they themselves emphasized trusting their veterinarian and having a good relationship with them:

“*The rapport and mutual trust built up with same vet over a number years has enabled continued care of chronic condition to be managed effectively and efficiently during C19 restrictions.”* (Respondent 287)

A small number of all respondents (but close to a quarter of those whose dogs required or was suspected of needing end-of-life care) described changing vets in order to be able to be with their dog during consultation:

“*I've left the vet I was with for 20 years and have joined another one that allows you to go in with your pet as long as you have a mask on which is what should have happened all along.”* (Respondent 600)

Respondents who were able to accompany their dogs within the practice also described changes in how procedures were carried out to enable them to accompany their dogs. These changes were more common for end-of-life treatments. The adaptations included the vet stepping away from the dog for part of the procedure, conducting consultations outside (e.g., in the car park), leaving the door open so owners could see the dog and using a long line to carry out a euthanasia procedure:

“*The vet and nurse carried her into the practice and inserted a cannula with a long line. They brought her outside onto a blanket where I could be with her, talking to her and holding her in the drizzle when she died. It was awful but I understand that it was the best they could do for us and they were so empathetic.”* (Respondent 51)

The most prominent sub-theme identified within the “Change in consultation setting” theme captures experiences of being unable to be with the dog during the consultation, which majority of the respondents found very distressing. Respondents thought that separation from them added to theirs and dog's stress:

“*Situation made even more stressful because I couldn't be with my dog when the vet checked her (she is a nervous dog).”* (Respondent 542)

“*It was dreadful because I couldn't be with her. I feel I let her down by not being there at the end.”* (Respondent 705)

Approximately a third of respondents who experienced outdoor consultations also worried about lack of privacy in these cases. This was especially salient among owners who sought end-of-life-care:

“*Dealing with the passing of a much loved family member was very hard during the pandemic as it felt heartless. Being stood in the car park in view of other people waiting to see the vet whilst my dog died was horrific. Didn't feel we got a chance to say goodbye. The vets assistants just came and took him away. Gave us a couple of leaflets and told us to ring them when we had made a decision on cremation etc.”* (Respondent 409)

#### Experience of Quality of Care

More than half of respondents who commented on this subject did not experience any change in the quality of care provided, despite numerous changes in how veterinary practices were able to operate:

“*My dog was receiving ongoing chemo for a condition which presented before covid. As covid appeared the way we accessed the vet changed, such as phone calls and dropping him off in the car park, but his care was excellent and I was always made to feel welcome. He probably received better care than humans as his treatment was not interrupted because of covid”* (Respondent 384)

However, the difficulties in booking appointments, delays in accessing care and being unable to seek regular check-ups, impacted on the experienced quality of care and dog's health among approximately a third of owners:

“*The pandemic caused delays in appointments for the specialist. It took 8 months for hip dysplasia to be diagnosed.”* (Respondent 539)

“*Seeked [sic] antibiotic treatment for ongoing otitis. Took my vet 10 days to dispense medication due to furloughed vets and staff. Ended up having emergency TECA resulting in permanent vestibular and neurology issues. Angry beyond belief.”* (Respondent 87)

Most of those negatively affected cared for dogs with chronic health issues, who were particularly reliant on regular check-ups to monitor dog's health. Some expressed feeling like they were left alone to monitor their dog's health and a small number said that they needed to wait for their dog's health to deteriorate to access care:

“*It was very difficult. Prior to March 2020, our dog had regular blood tests at least monthly along with very close monitoring of his medication. Since March we have had no blood tests and pretty much left to sort his medication levels ourselves.”* (Respondent 447)

“*During the first lockdown we had to stop our dog's Cushings medication completely as our vet was only seeing emergencies. When we told the receptionist the medication could kill our dog without a blood test to check her levels we were told they would see her in an addisonian crisis or to put her to sleep (we later complained to the vet about the receptionist's attitude) but as a blood test isn't an emergency they weren't allowed to do it. We had to stop the medication altogether and wait for the Cushings symptoms to return, by which point the lockdown restrictions had relaxed a bit and we were able to get our dog tested.”* (Respondent 524)

In addition, a combination of telemedicine, being unable to accompany a dog within the practice, face coverings and consultations being conducted outdoors, resulted in challenges in client-vet communication, reported by approximately a quarter of respondents:

“*The lack of face to face consultation resulted in me not knowing how ill she was. If I had known I would have been more insistent on her treatment.”* (Respondent 573)

“*Working with the vet was so hard as I had to try to describe her symptoms over the phone and email, with photos and video, and not have the reassurance that I was doing a good enough job. It was new ground for all parties.”* (Respondent 339)

Another dog owner who believed that their dog died as a result of disruptions to their care caused by the pandemic, emphasized the impact of being unable to accompany dog into the practice:

“*If covid was not happening after my dogs mouth continued to bleed for several days I feel more would have been done if I had been able to speak to the vet in the practice like normal but again the dog was taken off me and then brought back with some antibiotics”* (Respondent 318)

#### Impact on Owner's Finance

A small number of owners in this study (mostly those carrying for dogs with chronic health issues) said that COVID-related restrictions had a negative impact on their finance. This was due to vet practices increasing the cost of consultations, practices restricting their operating hours (meaning that more consultations were treated as out-of-hours and charged at a higher rate), owners needing to seek care outside of their regular practice or having to travel further than usual as their regular practice was closed or unable to offer appointments:

“*Due to the elderly age of my dog with underlying chronic conditions, seeking regular veterinary treatment and monitoring is key for my dogs to continued health and happiness as can be! Covid 19 restrictions has impacted the services my [veterinary charity] can offer (emergencies only), which in turn severely effects my personal finances seeking an alternative private vet. My dog's conditions are very costly to treat and monitor, but essential to [their] quality of life. I am looking forward to [charity] treatment limitations from Covid to be lifted.”* (Respondent 363)

“*I have been disappointed with local vets who have increased prices and won't allow face to face covid safe appointments (…)”* (Respondent 222)

“*My Veterinarian was not open for treatment, so we had to travel 20 miles for treatment at an emergency vet hospital.”* (Respondent 268)

### Reasons for Not Seeking Veterinary Healthcare During the Pandemic

The main reasons for not seeking or being unable to access care are summarized in Table 6. Typically owners did not seek care because vets were only seeing emergencies and because the owner did not want the dog to go in alone. Fear of contracting COVID-19, feeling unwell with COVID-19 symptoms or shielding were rarely listed.

**Table 6:** Reasons for not seeking/ being unable to access care for different health issues; *n* (%).

**Type of issues/ Reason**	**Acute** ***n* (%)**	**Preventative healthcare** ***n* (%)**	**Chronic** ***n* (%)**	**End-of-life-care** ***n* (%)**
Dog's health improved	7 (12)	1 (2)	3 (5)	4 (44)
Fear of contracting COVID-19	2 (3)	2 (4)	1 (2)	0
More precautious financial situation	1 (2)	12 (21)	0	1 (11)
Vets were only seeing emergencies	27 (44)	4 (7)	25 (42)	0
Vets assured it's ok to miss out treatment	3 (5)	14 (25)	4 (7)	0
I found out it's ok to miss out treatment	1 (2)	8 (14)	1 (2)	0
Picked up medications from the vets/ordered online	0	2 (4)	0	0
Found advice online	1 (2)	1 (2)	2 (3)	0
Used home remedies	3 (5)	0	4 (7)	0
Used leftover medications	4 (7)	0	2 (3)	0
Didn't want the dog to go alone	8 (13)	11 (19)	16 (27)	3 (33)
Dog's behavior is difficult to manage especially with social-distancing	4 (7)	2(4)	2 (3)	1 (11)
TOTAL	61 (100)	57 (100)	60 (100)	9 (100)

Qualitative analysis helped to understand factors owners considered when deciding whether to seek care during the pandemic and reasons for not seeking care. These are summarized below by reviewing the main themes: “Deciding to seek care,” “Fear of dog being alone,” “Coping with dog loss.”

#### Deciding to Seek Care

The vast majority of respondents stated that pandemic had no impact on how they made a decision to seek care. Owners reported seeking veterinary care for acute health issues when home treatments did not work, when the dog's condition deteriorated or was not improving. Almost all owners said they would seek care if they thought their dog was ill, in pain (i.e. for acute issues) or their life was at risk:

“*She was in pain and we had taken usual treatments such as cleaning and antihistamine.”* (Respondent 67)

A small number of respondents sought preventative healthcare because their insurance was dependent on continuity of treatment, for a primary vaccination for a new puppy and to make sure their dog's vaccination was up-to-date in case they needed to be hospitalized and their dog needed to be taken care of by someone else::

“*It is important to keep up to date on vaccinations for health and insurance.”* (Respondent 64)

“*She was a puppy who needed 1*^*st*^ *vaccinations, had to be done.”* (Respondent 79)

“*[T]he pandemic which was what led to feeling the extra need around vaccinations being brought up to date in case we many potentially have both required hospitalisation had we both been hospitalised due to Covid 19 when the risks around the virus became understood to realise that could potentially increase the risks of all pet carers becoming potentially hospitalised at once.”* (Respondent 232)

Approximately a quarter of those who shared their views on this subject described considering whether veterinary care is necessary, or could be avoided (e.g., looking for relevant treatment options online or on social media, or by contacting the vet on the phone first):

“*Due to the pandemic I'm using my own herbal & homeopathic remedies and dietary supplements ie turmeric etc, to help until I can get her in to see the vet accompanied by me. If pain increases I'll take her and hand her over to be seen without me. Without the pandemic restrictions she would have seen a vet already.”* (Respondent 366)

“*Care was not urgent so was postponed until after lockdown due to advice from the vet.”* (Respondent 344)

A small number of respondents additionally considered how easy it is to travel to the vet:

“*Travelling to the vets was more difficult as we do not own a car and could not ask friends”* (Respondent 5)

More than a quarter of respondents also described a delay in seeking care or not seeking care being caused by uncertainty if their dog needed veterinary support:

“*The pandemic has certainly made me wait to call the vet rather than call straight away. The most recent bout of diarrhoea should probably have been seen about at least a few days before we called. I feel bad about leaving it but didn't want to cause more work for the vets when it's so difficult at the moment.”* (Respondent 663)

Only a small number of owners reported considering the COVID-19 related risk (to themselves and the veterinarian) when seeking care:

“*I considered whether an appointment was required before attending site. I considered how many people could enter. I considered what may be needed when attending to ensure minimal visits. I considered the potential impact swallowing an object could have on my dog if medical advice was not sought.”* (Respondent 315)

#### Fear of Dog Being Alone

Across all health conditions (acute/ preventative healthcare/ chronic and end-of-life care), the main reason for not seeking care or delaying seeing care, expressed by approximately three quarters of those who commented on this subject, was not wanting a dog to go to the vet alone. This was particularly the case among owners who were considering euthanasia and among those whose dogs were fearful of vets:

“*I would have taken dog for vaccinations if I could stay with dog during appointment. I decided risk of disease is less likely than distress at dog being taken from me.”* (Respondent 335)

“*Altho (sic) this was handled sympathetically, we thought about (dog's name), getting progressively more paralysed and incontinent, and decided not to have her pts (put to sleep) until COVID restrictions allowed us both to be in the surgery with her (…) we perhaps let her go on for too long, we have no regrets.”* (Respondent 86)

A small number of respondents did not want their dog to go to vets unaccompanied, because they were not confident that their dog would be handled in a stress-free way:

“*I expect to be able to support my pet by being with them with a mask for safety. I have been able to do this at my vets. If this option was not available I would consider whether I needed to attend (…) I believe vets have a long way to go to understand pet handling needs and stress less handling.”* (Respondent 202)

#### Coping With Dog Loss

A handful of owners who had a recent experience of having to euthanise their dog also considered whether they can cope with another loss during the pandemic:

“*I had had a very traumatic time with having one dog severely attacked and having to rehome another dog and I couldn't face losing another dog.”* (Respondent 615)

### Predictors of Seeking Care and Urgency to Seek Care

#### Predictors of Seeking Care for Any Health Issues

Multivariable logistic regression model for seeking care for any health issue (${\text{X}}_{\left(5\right)}^{2}$ = 38.9, *p* < 0.001, R^2^ = 0.1, *n* = 695) is shown in Table 7. The model accurately categorizes 65% of those who reported intention to seek care (C-statistic= 0.65), and Hosmer-Lemenshow *p* = 0.2, indicating a good model fit. The odds of seeking care (compared to considering and deciding against or not considering it) were marginally lower for those who scored higher on MDORS emotional closeness sub-scale, and for dogs who were previously diagnosed with a chronic condition. The odds of seeking care were higher with a higher score on urgency to seek care questions and with a self-efficacy construct score.

**Table 7:** Multiple logistic regression model of seeking veterinary healthcare during the COVID-19 pandemic.

**Variable**	**Odds (95% CI)**	***p*-value**
MDORS emotional closeness sub-scale	1.0 (0.94–1.0)	0.05
Dog diagnosed with a chronic condition (comparison: not diagnosed)	0.5 (0.3–0.7)	<0.001
Urgency to seek care (total)	1.1 (1.0–1.1)	0.005
Self-efficacy (total)	1.1 (1.0–1.2)	0.009

#### Predictors of Urgency to Seek Care for Any Health Issues

The remaining two models assessed urgency to seek care (above mean score on urgency to seek care questions compared to below). The model fitted for the whole dataset (${\text{X}}_{\left(5\right)}^{2}$ =69.5, *p* < 0.001, R^2^= 0.12, *n* = 712, Table 8) shows that the odds of above mean urgency to seek care were positively associated with score on MDORS closeness scale, Perceived benefits construct score, Perceived susceptibility construct score and owners stating they were vulnerable to COVID-19. Above mean urgency to seek care was negatively associated with MDORS Perceived Cost scale; as this scale is reverse-scored, odds of higher urgency to seek care are associated with lower perceived cost of dog ownership. The model accurately categorized 60% of those who reported intention to seek care (C-statistic = 0.60), and Hosmer-Lemenshow *p* = 0.4, indicating a good model fit.

**Table 8:** Multiple logistic regression model of urgency to seek care given symptoms that could indicate common chronic health conditions.

**Variable**	**Odds (95% CI)**	**p-value**
MDORS emotional closeness sub-scale	1.04 (1.01–1.07)	0.006
MDORS perceived costs sub-scale	0.92 (0.89–0.96)	<0.001
Perceived susceptibility construct (total score)	1.14 (1.03–1.26)	0.013
Perceived benefits construct (total score)	1.07 (1.01–1.13)	0.019

Model sub-setted to the data of dogs with chronic health issues (${\text{X}}_{\left(5\right)}^{2}$ =65.4, *p* < 0.001, R^2^= 0.23, *n* = 350, Table 9) shows that the odds of above mean urgency to seek care were associated with an increasing score on MDORS closeness and interactions sub-scales, Perceived susceptibility and severity constructs scores. As in the earlier model, above mean urgency to seek care was negatively associated with MDORS cost scale, indicating that those who see costs of dog ownership as low seek care more urgently than those who see the cost as high). The urgency to seek care was associated with lower score in response to the statement “My dog's health has deteriorated since the beginning of the pandemic,” showing that those who generally disagreed were less likely to say they would seek care urgently. Model accurately categorizes 61% of those who reported intention to seek care (C-statistic = 0.61), and Hosmer-Lemenshow *p* = 0.4, indicating a good model fit.

**Table 9:** Multiple logistic regression model of urgency to seek care given symptoms that could indicate common chronic health conditions performed on the subset of dogs with chronic health condition diagnosis.

**Variable**	**Odds (95% CI) not scaled**	**p-value**
MDORS cost sub-scale	0.92 (0.87–0.98)	0.01
MDORS shared interactions sub-scale	1.07 (1.00–1.15)	0.001
MDORS emotional closeness sub-scale	1.07 (1.02–1.12)	0.04
Perceived susceptibility construct	1.22 (1.06–1.41)	0.002
Perceived severity construct	1.23 (1.07–1.41)	0.02
My dog's health has deteriorated since the beginning of the COVID-19 pandemic	0.75 (0.63–0.90)	0.003

### Future Plans

Qualitative analysis showed that, overall, respondents did not intend to alter their future healthcare plans because of their pandemic experiences. In fact, most owners of dogs with chronic conditions (as well as any other health issues) wished to continue to visit their veterinarian for regular, periodic healthcare checks, vaccinations, flea/ deworming treatments, and to weigh their dogs, when needed. Owners of dogs with chronic conditions also stated that they wish to regularly test their dogs and to attend specialist clinics to monitor their dogs and any impacts of medications (and in some cases, to carry out the tests that were unavailable during the pandemic):

“*I'll continue as normal, annual check ups and as and when required.”* (Participant 720)

Owners who relied on complementary therapies (physiotherapy and hydrotherapy in particular) were very keen to access these as soon as possible. A small number of owners with dogs with behavioral issues stated they wished to socialize their dogs to the veterinary practice when restrictions are lifted and to continue with behavioral management plans.

Few respondents stated that the pandemic made them reflect on their relationship with the veterinary healthcare team and that, from now on, they wish to be less dependent on them s:

“*COVID has enabled me to become less reliant on vets and to take on more responsibility for my dog's health myself. Hopefully this will lead to less consultations required throughout the year*.” (Respondent 726)

## Discussion

This study aimed to explore impacts of the COVID-19 pandemic on dog owners seeking veterinary healthcare in the UK, focusing in more depth on the experiences of owners caring for dogs with chronic health conditions. Although most of those who responded were able to access veterinary healthcare, delays in appointment availability and changes in how consultations were run had a disproportionate impact on dogs with chronic conditions, who rely on regular veterinary care to monitor and manage their health. Dog-owner relationship, owner's vulnerability to COVID-19 and owner's urgency to seek care given symptoms that could indicate common chronic conditions assessed through response to a hypothetical scenario were associated with veterinary healthcare seeking behavior during the pandemic. In addition, constructs derived from the Health Belief Model: self-efficacy in relation to seeking healthcare, dog's perceived susceptibility to illness, perceived benefits of seeking care, and perceived severity of the condition predicted urgency to seek care. Below we discuss our findings within the context of previous research.

### Experiences of Caring for Dogs With Chronic Health Problems Before and During the Pandemic

Owners of dogs with chronic conditions reported adapting their home and lifestyle to care for their pets. Caring for dogs with chronic conditions often involved frequent veterinary consultations. Whereas owners of dogs without such diagnosis reported seeking veterinary healthcare when required (i.e., in the case of emergency) and for annual health check-ups where preventative healthcare is provided, those caring for dogs with chronic conditions relied on their veterinarian to monitor their pet's health and provide ongoing treatment. In addition, owners of dogs with chronic health problems utilized a range of complementary treatments, supplements and medications to maintain their dog's health, which often requires substantial financial commitment. Our findings echo previous research, which shows that, if they can, owners of dogs with chronic conditions adapt their lifestyle, modify their home and seek non-prescription therapies to support their dog's health (28, 53, 54).

Our study found that during the pandemic, owners were often forced to change care routines for their pets: some reported that they could not take their dog for usual walks and those caring for dogs with chronic conditions reported that their pet's health suffered due to lack of access to physiotherapy or massage-therapy. Previous research demonstrates that caring for dogs with chronic conditions can lead to caregiver burden, which in turn is associated (possibly causally) with the owner's stress, depression and lower quality of life (53, 55, 56). For example, caring for a dog with osteoarthritis, among the most common health issues enumerated in this study, has previously been described as possibly contributing to owners feeling socially isolated and sometimes reporting difficulties in receiving a respite (28). Past studies show that difficulties in adhering to a pet's care routine correlate with higher caregiver burden (53). Therefore, given the challenges in maintaining a pet's routine reported in our study, the pandemic is likely to have worsened the caregiver burden and had a negative impact on the mental health of owners of dogs with chronic health issues in particular. Whilst delivery of ongoing treatment (such as chemotherapy or injections aimed to manage skin conditions) was rarely disrupted in this study, a large proportion of owners stated that they were unable to consult with their vet to monitor their dog's health. A small number of owners stated that this led to a deterioration of their dog's health and a large proportion explained that this was a cause of anxiety, isolation and a sense that they are alone in making decisions about their pet, further highlighting how pandemic-related restrictions to accessing veterinary healthcare may have contributed to caregiver burden. To better support all dog owners, we recommend that any future restrictions classify veterinary consultations aimed at monitoring chronic illness, as well as complementary therapies with proven efficacy, as essential work. Greater awareness of the importance of movement for dogs with chronic conditions is also needed.

### Experiences of Seeking and Accessing Veterinary-Healthcare During the Pandemic and Reasons for Not Seeking Care

Similar to other studies (12, 13, 57), we identified that a number of dog owners struggled to book non-emergency veterinary appointments and appointments with non-veterinary healthcare providers, which disproportionally affected owners of dogs with chronic health conditions. The vast majority of those who responded to our online survey were able to access a consultation for acute, preventative or chronic health issues and end-of-life care when needed; however, this may not have been the case for all UK pet owners.

Our study found that owners who needed to access preventative care were significantly more likely not to seek it, were unable to access it, and those who needed end-of-life care were significantly more likely not to seek it, compared to other health issues. This suggests that previously identified pandemic-related delays in provision to preventative care (13) may have been due to changes in owners' healthcare veterinary seeking behavior as well as actual accessibility of services. Whilst short-term delays in preventative care are unlikely to have a negative impact on the welfare of otherwise healthy dogs (58), delaying euthanasia has been identified as a serious welfare concern (59). Previous findings suggest that when dog owners refuse or delay euthanasia, palliative treatment is provided to protect animal welfare (60). The manner in which information about life-limiting conditions in dogs is communicated is vital to ensure owner understanding of dog's health (61). To be effective, communication about end-of-life care should be direct, delivered in multiple ways, in clear language, in an unrushed and ongoing manner, i.e., enabling owners to ask additional questions after the consultation (61). It is unlikely that these conditions were easy to achieve during the pandemic, possibly impacting upon owner's decisions regarding euthanasia and palliative care options. This further corroborates the impact of delays in seeking end-of-life care on animal welfare. In addition, a small number of owners delayed end-of-life care for their dogs as they feared they would not be able to cope with pet loss during the pandemic. Whilst this is also a likely influence on delayed euthanasia pre-pandemic, general deterioration of dog owners' mental health during the pandemic (62) and the importance that pets played in maintain owners' wellbeing during the pandemic (20–25, 63–65) is likely to have made this factor more pertinent.

Although close to half of those who needed care for an acute/preventative issue were unable to accompany their dogs into the clinic, the majority of those who sought end-of-life care were able to, which demonstrates that many clinics worked hard to ensure that owners could be present with their dogs during the consultations. Respondents described a number of ways in which veterinary practices adapted to enable owners to be present with pets, e.g., by carrying out consultations outdoors, using a long line for euthanasia (enabling the vet to carry out the procedure in a socially-distant manner), or by altering the protocol so that vet and owner were taking turns in being near the dog. This shows that the interpretation and enactment of the official guidelines for social-distancing and COVID-19-safety (1, 7, 66) was not fixed, but involved developing protocols and practices (67) that worked within the local environment. Most owners in our study were grateful for this opportunity to be with their dog; however, some found lack of privacy during outdoor consultations difficult. This could be ameliorated by using privacy screens when carrying consultations outside of the clinic and having baskets ready with items such as tissues for the owner and a small cloth bag for pet's collar or container to place hair clippings in (68). Access to grief resources (69) and having a veterinary social worker on staff for follow-up calls and to care with potential compassion fatigue as experienced by veterinary team members is also advisable (70).

The vast majority of respondents showed high levels of trust in their veterinarian's skills and valued their opinion. Some owners reported that they were happy for their dog to be seen without them as they trusted their vet's opinion and handling skills. In addition, respondents generally trusted in their vet's reassurance that postponing seeking care would not affect their dog's welfare. However, the most common reasons for not seeking care, or delaying access, was being unable to accompany a dog into the practice and uncertainty if care was available. Separation of the dog and owner impacts on likelihood and timing of healthcare seeking and dog's distress during consultations (71, 72). Owners preferred to be present with their dog in order to manage their behavior and some worried about their veterinarian being able to handle their dog in a stress-free manner, in particular if a dog was already anxious. Many owners who sought end-of-life care switched healthcare provider to one who allowed them to be present during the procedure. These findings reflect previous research which showed that being unable to accompany dogs into the practice was stressful and resulted in delays in seeking care (12, 13). Veterinary practices should therefore strive diligently to enable owners to accompany their pets during consultations wherever possible. Our findings also add weight to the value of socializing dog to the formal handling and within veterinary clinics and show that veterinarians' stress-free handling skills are central to building trust in owner-vet relationship.

Our study echoes previous findings in showing that COVID-19-related restrictions impacted on owner-vet communication (13, 17). As clear communication with veterinary clients emerged as important in maintaining and continuity of care when normal operating protocols are disrupted, veterinary training in this area should be extended to communication via telehealth. Additionally, although a minority of respondents were considered lower income, this study nevertheless identified that changes in how care was provided could impact owner's finances. Owners who previously relied on subsidized treatments offered by veterinary charities were particularly affected, as access to their regular (subsidized) veterinarian was not available and seeking healthcare privately sometimes meant paying out of hours fees and traveling further to access healthcare. In our sample, only a small number of respondents pointed this out as a problem. Lack of accessible pet-friendly transport, in addition to financial constraints, is a barrier to veterinary healthcare seeking within underserved and marginalized communities which were under-represented in our convenience sample. This echoes the pattern identified in the USA that shows that owners from most underprivileged backgrounds were potentially most affected by changes in care provision for their pets during the pandemic (14, 15).

Finally, there was no significant difference in the number of veterinary visits before and since the pandemic for dogs with a chronic health issue diagnosis; dogs without this diagnosis visited their veterinarian significantly more often since the pandemic began than before. This result should be interpreted cautiously. The observed pattern could reflect an annual variation in the number of vet visits [which are known to peak in spring and dip over winter; (73)]. Owners may have also noticed more health problems as a result of spending more time with their dogs during the pandemic. In this survey, we did not define what constitutes a vet visit, therefore it is possible that this increase can be attributed to contacting a vet using telemedicine. Finally, owners who visited their veterinarian recently during the pandemic may have been more inclined to complete our survey.

Overall, the pandemic did not change how majority of owners in our study intend to engage with veterinary services in the future. However, a small number of respondents believe that they can now be more independent of their vet and take on more veterinary tasks themselves. They may have also switched veterinary practices during the pandemic because of perceived poor experiences and because they were able to. A few respondents may have lasting impacts due to how euthanasia was handled.

### Using Health Belief Model to Predict Intentions and Urgency to Seek Care

Strong associations between the behavior of seeking care and score on the urgency to seek care shows that owners who report high urgency on hypothetical scenarios likely apply a similar rule when deciding if their dog needs to seek care in real life. Self-efficacy to seek care was identified as an important predictor of seeking veterinary care in other contexts, such as adherence to elimination diet trial (74), showing that improving dog owners' health literacy, i.e., dog owner's ability to seek, evaluate, and apply knowledge regarding dog health, could increase their engagement with veterinary care. Changes in how care was delivered and difficulties in accessing care for non-emergency conditions identified in the qualitative analysis could lead owners of dogs with chronic health conditions to delay seeking care, reflected here. The effects of the owner's relationship with a dog on odds of seeking care was very weak, albeit significant. This finding may reflect that those who reported being emotionally closer to dogs were more likely to delay seeking care due to anxiety related to being separated from the dog during the consultation, which emerged as an important qualitative theme.

Our findings show that urgency to seek care for chronic conditions is driven primarily by the strength of relationship with a dog and HBM constructs. Previous studies utilized the HBM in exploring factors related to seeking healthcare focused primarily on seeking vaccinations and preventative care (33, 34, 74). Our study shows that HBM constructs are useful in predicting healthcare seeking for chronic health issues. None of the owner's or dog demographic variables, or household-related variables (such as income or fear of income loss due to pandemic) were significant predictors of intentions to seek healthcare or urgency to seek care, similar to findings reported by Park et al. (75) but at odds with other findings which identified that engagement with preventative healthcare could be predicted from owner demographic variables (33). COVID-19 variables were also not identified as significant predictors of seeking care. The strength of the relationship with the dog has also been previously identified as important factor predicting veterinary healthcare seeking (33), owners' likelihood of seeking healthcare for themselves, should they be infected with COVID-19 (24), further emphasizing the importance of “one health” approach to veterinary and human medical care. Our findings suggest veterinary practices could draw on the dog-owner relationship when designing communication and interventions that aim to encourage veterinary healthcare seeking. In addition, clear communication around severity of the condition and dog's susceptibility to it, and bolstering the owner's perception of efficacy in management of dog's health and healthcare seeking, could improve urgency to seek care.

### Study Limitations

Data for this study was collected retrospectively, therefore comparisons with the pre-pandemic care routines and interactions with veterinary healthcare need to be interpreted cautiously, as these reports may have been affected by recall bias. The survey format enabled collection of both qualitative and quantitative data, but compared to other methods of data collection (e.g., in-depth interviews), the richness of qualitative data is somewhat limited, as for example follow-up questions cannot be asked for deeper exploration of an issue. During the period when the survey was open for completion, another lockdown in England was introduced (from January 2021); this was not captured by our data. Although care was taken when advertising the survey to recruit a diverse range of owners (e.g., geographic location/ breed/ health issues), response bias cannot be precluded. The survey was completed by 70% of those who started it. It is unclear whether those who started the survey differed with respect to their experiences from participants who completed the study, which may have further contributed to a response bias. It is possible that owners who had particularly strong opinions about veterinary care, or during the pandemic, were more likely to complete the survey than those with more moderate views.

The results of multivariable regression models need to be interpreted cautiously. The c-statistic in all three models is relatively low (0.6–0.65), meaning that models can accurately classify 60–65% of outcomes. In addition, the pseudo R^2^ statistic in all three models is also low (0.1–0.23), meaning that <23% of the variability in the dependent variables (seeking care and urgency to seek care) can be predicted from these models and suggesting model under-fitting. Although a very high R^2^ may indicate model over-fitting and poor generalisability, our results suggest that all three models have a relatively low predictive power and accuracy. Unfortunately, low R^2^ (<0.5) are common in human-animal interactions research, possibly due to the complex nature of these relationships and measurement-related challenges (76). Low R^2^ values may additionally reflect a large number of additional factors possibly associated with the outcome variables that were not measured here. These factors could include, for example, the owner's personality, access to transport, digital literacy skills and access to subsidized veterinary healthcare. These factors warrant further investigation in the future. We decided to include these models in the current publication despite their limitations as the area of veterinary healthcare seeking is understudied and therefore even limited models may aide future studies.

Like most human-dog-interactions-related research (77), our sample was biased toward well-educated women with above median income and therefore was not representative of the broader UK dog-owning population. This limits the generalisability of our findings and recommendations. COVID-19 pandemic exasperated structural inequalities within the UK: compared to prosperous areas, the most deprived areas of England suffered more than twice as many deaths from COVID-19; ethnic minorities and those with pre-existing disabilities were more likely to die and suffer post-infection complications (78, 79). Despite government interventions, a near-decade of austerity measures that preceded the pandemic in the UK meant that the incomes of the lowest-earning households and those working on zero-hours contracts were significantly more affected by the pandemic than incomes of those on higher salaries and on permanent contracts (80), adding to food (81, 82) as well as fuel poverty (83, 84) and possibly squeezing the budget available for pets' healthcare. Although our study did not identify any associations between income or income concerns and healthcare seeking, it is likely that the characteristics of our sample made it impossible to detect this effect. Barriers to accessing veterinary healthcare identified in this study were most likely far greater among those most heavily impacted by the pandemic and those experiencing financial pressures. Owners on low income may have also been unable to change the veterinarian when their veterinarian was not taking appointments or to travel further to access veterinary care. Moreover, prior to the pandemic 22% of the UK's population lacked basic digital skills (85), needed when booking veterinary appointments or using telemedicine, (as many practices required using a phone or tablet-based application for this purpose). As the access to the internet is strongly related to household income [with just 51% of households earning between £6,000–10,000 able to access the internet compared to 99% of households on income above £40,000; (86)], it is likely that for the most financial disadvantages dog owners reliance on telemedicine was a further barrier to veterinary healthcare. Therefore, the impact of the pandemic on the owner's finance and the role of finance on owner's ability to care for their dog warrants further exploration using different tools of data collection that enable stratified or random sampling. Ten percent of the UK's dogs are not registered with a veterinarian (87). As most of this study participants visited their veterinarian regularly, this research does not inform about the impact of the pandemic on the health and welfare of dogs who do not receive regular veterinary care.

Human (and consequently pet) health is affected by multiple layers of interrelated factors, including individuals' biological characteristics (e.g., their genetics) and lifestyle, but also structural factors. These include individual's social and community networks (through which dog owners may, for example, seek information or help), living and working conditions (including employment, the structure of healthcare service, housing) and general socio-economic, cultural and environmental conditions, which may encompass national policies regarding veterinary care (88). Constructs derived from the Health Belief Model use in this study help to highlight ways in which individual behavior can be changed to encourage seeking veterinary healthcare. However, this approach does not account for structural influences that impact on veterinary healthcare seeking, including structural inequalities outlined earlier. The individual-based approach may also place undue emphasis on individual responsibility to improve their access to veterinary healthcare without highlighting structural changes (88, 89) that may be needed to ensure serving all socioeconomic groups, including those who cannot travel to access their veterinarian, those unable to use telemedicine and those on low income. This study did not explore the nature of structural changes needed, however it is plausible that subsidized or free-of-charge, mobile and face-to-face veterinary care may play an important role.

Finally, studies show that the veterinary community across the globe was under immense pressure to delivery care during the pandemic, with many veterinarians feeling under-valued, experiencing more stressful moments at work, struggling to communicate with clients and experiencing lower levels of mental wellbeing compared to before the pandemic (16–18, 90–92). Our study did not explore their important experiences.

## Conclusions

This study shows that during the COVID-19 pandemic in the UK, veterinary practices managed to support the needs of the vast majority of those seeking urgent care and accommodated most of those looking for preventative care, for appointments to monitor dog's chronic conditions and for end-of-life care. Some veterinary practices worked creatively to adapt the way appointments were delivered to enable dog owners to be present during consultations in a COVID-19-secure way. However, this was not always possible and led some owners to delay seeking preventative care, euthanasia and for chronic health conditions, and in some cases resulted in traumatic experiences. Owners of dogs with chronic health issues, who relied on regular consultations for monitoring conditions, listed delays in accessing veterinary healthcare and complementary therapies, impacts of COVID-19-related restrictions on client-vet communication and being unable to accompany a dog during a consultation, as reasons for deterioration in their dog's health. The main predictors of seeking care and urgency to seek care were the dog-owner relationship and Health Belief Model constructs. This suggests that individual-level behavior interventions aimed at promoting veterinary healthcare seeking could include targeting attitudes related to benefits of seeking care, promoting health literacy and self-efficacy and capitalizing on the dog-owner bond. Seeking veterinary healthcare during the pandemic was also associated with higher costs, which is particularly problematic for owners who rely on subsidized services or who may find themselves needing to in the future. Further consideration toward affordability of care is needed (93), in particular in the light of a growing population of dogs in the UK, raise in costs of living and reported shortages of veterinary staff. Future population-level interventions aimed at improving access to veterinary care needs to consider how costs of care may affect the decisions of the most underprivileged owners, particularly those caring for dogs with chronic conditions. Risk of COVID-19 transmission was rarely cited as a reason for not seeking care and seems to have little impact on owners' decision-making. Finally, the pandemic did not seem to impact future healthcare plans of the majority of dog owners who responded to our online survey.

## Data Availability Statement

The raw data supporting the conclusions of this article will be made available by the authors, without undue reservation.

## Ethics Statement

The studies involving human participants were reviewed and approved by Ethical Approval Application number: VREC1044. The patients/participants provided their written informed consent to participate in this study.

## Author Contributions

SCO-G, TF, TMG, DAS, LW, and CW: conceptualization, methodology, and writing—original draft. SCO-G and IL: formal Analysis. SCO-G: data curation. SCO-G, TF, TMG, and CW: writing—review and editing. SCO-G and CW: funding acquisition. All authors contributed to the article and approved the submitted version.

## Funding

This study was funded by the Animal Welfare Foundation (AWF). AWF is a fundraising and grant giving charity (charity number 1192203) directed by veterinary and animal welfare professionals, which uses veterinary knowledge to improve the welfare of animals through science, education and debate. More information can be found at www.animalwelfarefoundation.org.uk.

## Conflict of Interest

The authors declare that the research was conducted in the absence of any commercial or financial relationships that could be construed as a potential conflict of interest.

## Publisher's Note

All claims expressed in this article are solely those of the authors and do not necessarily represent those of their affiliated organizations, or those of the publisher, the editors and the reviewers. Any product that may be evaluated in this article, or claim that may be made by its manufacturer, is not guaranteed or endorsed by the publisher.
