# Supplementary material for: Impacts of COVID-19 on Owner's Veterinary Healthcare Seeking Behavior for Dogs With Chronic Conditions: An Exploratory Mixed-Methods Study With a Convenience Sample
Source: Front Vet Sci. 2022 May 26;9:902219. doi: 10.3389/fvets.2022.902219 (PMC9204258; doi:10.3389/fvets.2022.902219)
Supplement: Supplementary file 1 [file Table_1.pdf]

Supplementary material

Appendix A. Summary of questions regarding seeking care for acute, preventative, end-of-life, chronic health issues, attitudes to healthcare seeking and urgency to seek care.

Questionnaire Section	Questions	Response options (recoding pattern)
**About your dog**	Your dog is a:	Male<br><br>Female
	How old is your dog?	Number (years recoded to months)
	When did you get your dog?	Before COVID-19 pandemic (before 23rd of March 2020)<br><br>Since COVID-19 pandemic (after 23rd of March 2020)
	What breed is your dog?	List + 'Other, please specify'
	What size is your dog?	Toy (e.g. Chihuahua)<br><br>Small (e.g. Terrier)<br><br>Medium (e.g. Colie or Spaniel)<br><br>Large (e.g. Labrador or German Shepherd)<br><br>Giant (e.g. Great Dane)<br><br>Recoding pattern: small (toy/ small), medium (medium), large (large/giant)

	Where did you acquire your dog?	Commercial or hobby breeder<br><br>I bred the dog myself<br><br>UK dog shelter/ Rescue organisation<br><br>Overseas dog shelter/ Rescue organisation<br><br>Online<br><br>Given to me by friend or family member<br><br>Service dog (e.g. a guide dog, hearing dog provided by the specialist charity)<br><br>Other<br><br>Recoding pattern: breeder (commercial or hobby breeder, I bred the dog myself), shelter or rescue (UK dog shelter/ Rescue organisation/ Overseas dog shelter/ Rescue organisation), Other (Online/ Service dog/ Given to me by friend or family member)
	Is your dog neutered (spayed/ castrated)?	Yes<br><br>No<br><br>Unknown
	Looking at the picture above, which set of circles best describes the relationship between you and your dog? (ref)	1-7<br><br>Recoding pattern: weak (1-3), medium (5-4), strong (6-7)

	MDORS questionnaire (Dwyer et al., 2006)	5- point Likert scale with at least once a day- never and strongly agree- strongly disagree as anchors
	In the year before the COVID-19 pandemic (prior to 23rd March 2020), how many times did your dog visit a veterinarian?	Number. For the purpose of the analysis, this was converted to a monthly average.
	Since the restrictions imposed due to the COVID-19 pandemic (from the 23rd March 2020), how many times in total did your dog visit a veterinarian?	Number. For the purpose of the analysis, this was converted to a monthly average.
**Veterinary care during the COVID-19 pandemic: acute care**	Did your dog potentially[1] need treatment for an acute[2] (sudden-onset) condition between the first national lockdown (23rd March) and now?[3]	No acute medical condition<br><br>Skin infection or other skin issues<br><br>Gastroenteritis (vomiting and diarrhoea)<br><br>Torn nail or dew claw<br><br>Open wound (including a dog bite)<br><br>Broken limb or tail<br><br>Joint/ ligaments problems (e.g. cruciate ligament tear, hip dysplasia)<br><br>Teeth injury<br><br>Swallowed objects<br><br>Heatstroke<br><br>Poisoning (e.g. eating chocolate/ plants)

		Eye infection<br><br>Ear infection<br><br>Urinary infection (bladder/ kidney)<br><br>Allergy<br><br>Fits/ tremors<br><br>Don't know/ can't remember<br><br>Other, please describe
**Veterinary care during the COVID-19 pandemic: standard preventative care**	Did your dog potentially[1] need standard preventative healthcare such as vaccination, flea or worming treatment, or neutering since the beginning of the restrictions imposed due to the COVID-19 pandemic (23rd March and now)?	No, the treatment wasn't required<br><br>Vaccination (primary or booster)<br><br>Flea treatment<br><br>Worming treatment<br><br>Neutering<br><br>Nail trimming<br><br>Don't know / can't remember<br><br>Other, please describe
**Veterinary care during the COVID-19 pandemic: end-of-life-care**	Did your dog potentially[1] require end-of-life care (i.e., euthanasia or being 'put to sleep') sometime during the COVID-19 pandemic (i.e., from the23rd March2020 and now)?	Yes, I wish to continue with this section<br><br>Yes, but I want to skip other questions in this section<br><br>No, my dog did not require end of life care

**Veterinary care during the COVID-19 pandemic: chronic health conditions**[4]	Does your dog have a chronic health condition[4]?	Yes<br><br>No
**Veterinary care during the COVID-19 pandemic: chronic health conditions**	If your dog potentially needed care for more than one chronic conditions, please tell us about the earliest one	Osteoarthritis and other orthopaedic conditions<br><br>Epilepsy<br><br>Diabetes<br><br>Dental problems (periodontal disease)<br><br>Obesity<br><br>Skin problems<br><br>Cancer treatment<br><br>Glaucoma<br><br>Hypothyroidism<br><br>Dry eyes<br><br>Allergies<br><br>Irritable Syndrome/ inflammatory bowel disease<br><br>Pancreatitis<br><br>Kidney disease

		Liver disease<br><br>Endocrine disorder (e.g. Cushing's or Addison's disease)<br><br>Cognitive dysfunction<br><br>Degenerative heart disease<br><br>Don't know/ can't remember<br><br>Other, please describe
**Veterinary care during the COVID-19 pandemic: end-of-life-care**	Did your dog potentially require end-of-life care (i.e., euthanasia or being 'putto sleep') sometime during the COVID-19 pandemic (i.e., from the23rd March2020 and now)?	Yes, I wish to continue with this section<br><br>Yes, but I want to skip other questions in this section<br><br>No, my dog did not require end of life care
**Veterinary care during the COVID-19 pandemic: end-of-life-care**	What was the reason for considering end-of-life care? Please select all applicable options.	Cancer<br><br>Traumatic injury<br><br>Age-related poor health<br><br>Behaviour problems<br><br>Don't know<br><br>Chronic condition, please describe<br><br>Other, please describe
**Veterinary care during the COVID-19**	When did your dog potentially need treatment for the condition selected above?	During the first strict national lockdown (between 23rd March and 13th May 2020)

**pandemic for acute care, preventative care, end-of-life care and chronic care.**		After the first strict national lockdown measures were relaxed (between 13th May and<br><br>5th November 2020)<br><br>During a local lockdown/ in an area with local restrictions (e.g. classified as “tier 3”)<br><br>(between 13th May and 5th November 2020)<br><br>During the second strict national lockdown (between 5th November and 3rd<br><br>December 2020)<br><br>After the second national lockdown (after 3rd December 2020)
**Veterinary care during the COVID-19 pandemic for acute care, preventative care, end-of-life care and chronic care.**	Is the time period selected above accurate?	Yes, I’m sure of the date<br><br>No, I’m not completely sure of the date
**Veterinary care during the COVID-19 pandemic for acute care, preventative care,**	Did you seek veterinary advice, care or treatment since the beginning of the restrictions imposed due to the COVID-19 pandemic (23rd March and now) for [the acute (sudden-onset) condition] mentioned above?	Yes, I considered it, but at that time I decided against accessing veterinary advice, care or treatment<br><br>Yes, I sought to access veterinary advice, care or treatment<br><br>No, I did not consider seeking veterinary advice care or treatment at the time

**end-of-life care and chronic care.**		
**Veterinary care during the COVID-19 pandemic for acute care, preventative care, end-of-life care and chronic care.**	Were you able to access veterinary advice, care or treatment when potentially needing it?	Yes<br><br>No
**Veterinary care during the COVID-19 pandemic for acute care, preventative care, end-of-life care and chronic care.**	How was the care received (please choose all that apply)?	Me/another family member/friend and my dog visited the veterinary practice as usual/wearing facemask.<br><br>My dog was seen by the vet or vet nurse and treated outdoors, e.g. in the car park.<br><br>Due to COVID-19 related restrictions, I needed to hand my dog to the vet/ vet nurse who treated the dog in my absence.<br><br>Telephone advice was given instead of a face-to-face consultation.<br><br>Advice was given via an email or an app instead of a face-to-face consultation.<br><br>After a telephone, app or email consultation, my dog was seen in the veterinary practice.<br><br>I collected my dog’s medical treatment/ prescription or it was delivered to me, without the vet/ vet nurse seeing the dog.

		Other, please describe
**Veterinary care during the COVID-19 pandemic: end-of-life-care**	How was the end-of-life care handled? (please choose all that apply):	My dog and myself/ a friend or family member visited the veterinary practice as usual/ wearing a facemask and the dog was put to sleep.<br><br>My dog was put to sleep by the vet or vet nurse outdoors, e.g. in the car park.<br><br>Due to COVID-19-related restrictions, I needed to hand over my dog to the vet/vet<br><br>nurse and my dog was put to sleep in my absence or in the absence of a family member/friend.<br><br>My dog was put to sleep at home by the vet who visited us.<br><br>My vet and I decided not to put my dog to sleep at this time.<br><br>Other, please describe
**Veterinary care during the COVID-19 pandemic for acute care, preventative care and chronic care.**	Why were you unable or decided not to seek veterinary help or advice during the COVID-19 pandemic? Please select all applicable reasons.	Not applicable- I sought to access and were able to access veterinary care<br><br>Fear of contracting COVID-19.<br><br>My veterinarian was only seeing dogs in the case of an emergency at that time.<br><br>Difficulty in managing my dog behaviour and maintaining social distance.<br><br>I did not want my dog to be assessed by a vet/vet nurse without myself or another

		family member present.<br><br>My vet assured me that missing this treatment was unlikely to affect my dog’s long term<br><br>health.<br><br>I found out that missing the treatment was unlikely to affect my dog’s long-term health<br><br>without asking for a vet’s advice.<br><br>More precarious financial situation due to furlough/ redundancy<br><br>I obtained advice from the internet<br><br>I used home remedies<br><br>I used previous left-over medication<br><br>My dog’s condition improved without needing treatment<br><br>Other, please describe
**Veterinary care during the COVID-19 pandemic: end-of-life-care**	Why were you unable or decided not to seek veterinary help or advice during the COVID-19 pandemic? Please select all applicable reasons.	Not applicable- I sought to and was able to access veterinary care.<br><br>Fear of contracting COVID-19.<br><br>Difficulty in managing my dog behaviour and maintaining social distance.<br><br>I did not want my dog to go to the veterinarian unaccompanied.

		More precarious financial situation due to furlough/redundancy.<br><br>My dog's condition improved<br><br>Other
**Veterinary care during the COVID-19 pandemic for acute care, preventative care, end-of-life care and chronic care.**	Please explain factors you considered when deciding whether to seek care for the acute (sudden-onset) medical condition/ standard preventative healthcare/ end-of-life care for your dog was experiencing at this time. In what way was it similar or different from seeking veterinary care or treatment before the restrictions imposed due to COVID-19?	Open-ended
**Veterinary care during the COVID-19 pandemic: chronic care.**	In total, how many times did you visit a veterinarian for your dog's chronic health issues since the restrictions imposed due to the COVID-19 pandemic(from the23rd March 2020)?	Number. For the purpose of the analysis, this was converted to a monthly average.
**Veterinary care during the COVID-19 pandemic: chronic care.**	How long did the most recent veterinary consultation take? If you needed to travel for it, please consider the time needed to drive/ walk to the veterinarian and back and the time taken up by the consultation:	Number. For the purpose of the analysis, this was converted to a monthly average.
**Veterinary care during the COVID-19**	How do you manage your dog's chronic condition since the restrictions due to the COVID-19 pandemic have been imposed (from the 23rd March 2020)? How, if at all, is managing your dog's health during the COVID-19 pandemic different to how you managed it	Open-ended

**pandemic: chronic care.**	before? If your dog has been experiencing multiple chronic conditions, please tell us about the earliest one (the one you sought help for first)	
**Caring for dog's chronic health problems**	Did your dog have the chronic condition before the COVID-19 pandemic?	Yes<br><br>No, it was noticed and/ or diagnosed after the pandemic has started<br><br>My dog does not have a chronic condition
**Caring for dog's chronic health problems**	In the year before the COVID-19 pandemic (prior to 23rd March 2020), how many times did your dog visit a veterinarian for chronic health issues specifically?	Number. For the purpose of the analysis, this was converted to a monthly average.
**Caring for dog's chronic health problems**	Prior to the restrictions imposed due to COVID-19 pandemic, how long did the average veterinary consultation for<br><br>chronic health issues<br><br>take? If you needed to travel for it, please consider the time needed to drive/ walk to the veterinarian and back and the time taken up by the consultation. If your dog was experiencing multiple chronic conditions, please tell us about the<br><br>one that was first noticed	Open-ended

**Caring for dog's chronic health problems**	Is the veterinary treatment for the chronic health issues covered by your dog insurance?	Yes- completely<br><br>I pay the excess, but more than one half of the treatment cost is covered<br><br>I pay the excess and less than half of the cost is covered<br><br>No, it is not covered<br><br>No, my dog is not insured<br><br>Recoding pattern: at least partially covered by insurance (Yes-completely/I pay the excess, but more than one half of the treatment is covered/ I pay the excess and less than half of the cost is covered), not covered (No, it is not covered/ No, my dog is not insured)
**Caring for dog's chronic health problems**	How does the specific treatment/medications prescribed by the veterinarian affect your dog's behaviour?	Open-ended
**Caring for dog's chronic health problems**	In addition to the treatment/medications prescribed by the veterinarian, have you tried any other treatments? Please select all that apply	No other treatment/ therapy<br><br>Weight management<br><br>Home environment/ lifestyle adaptations<br><br>Modified exercise routine<br><br>Nutrition (changes in the main diet/ dietary supplements/ nutraceuticals)

		Homeopathic treatments<br><br>Herbal therapy<br><br>Acupuncture<br><br>Magnetic Field Therapy<br><br>Laser therapy<br><br>Hydrotherapy<br><br>Physiotherapy<br><br>Other
**Caring for dog's chronic health problems**	If your dog receives additional treatment/ therapy, how does the specific complimentary treatment affect your dog's behaviour?	Open-ended
**Caring for dog's chronic health problems (asked only if respondent stated that the dog has a chronic condition)**	The treatment and advice provided by my veterinarian is necessary to manage my dog's health condition.*<br><br>Interrupting the treatment would be very risky.*<br><br>I am aware of different treatment options for my dog's condition	5-point Likert scale with strongly agree- strongly disagree used as anchors<br><br>* Additional option: "Not applicable- I haven't contacted the vet or healthcare professional about my dog's chronic health issues"
**Knowledge and General attitudes to veterinary healthcare**	Please respond to the following statements:<br><br>I trust my veterinarian*<br><br>My veterinarian is knowledgeable*	5-point Likert scale with strongly agree- strongly disagree used as anchors<br><br>* These questions included an additional response option: "Not applicable- my dog is not registered with a vet"

	I care about my vet's views about how I manage my dog's health*<br><br>Managing my dog's behaviour during veterinary consultations is/has been difficult*<br><br>My vet thinks that providing my dog with regular check-ups or treatments is important*<br><br>Managing my dog's health is easy<br><br>I feel well informed and knowledgeable about my dog's health<br><br>My past experiences with veterinarians were not always good<br><br>I know where to seek information about my dog's health<br><br>I am confident in recognising when my dog is not feeling well<br><br>It's important to vaccinate dogs<br><br>I think that providing my dog with regular veterinary check-ups or treatments is important<br><br>My friends and family think that providing my dog with regular check-ups or treatment is important<br><br>I care about my friends and family's views about how I manage my dog's health<br><br>My dog's health has deteriorated since the beginning of theCOVID-19pandemic	

	If I needed it, I would be able to access veterinary care for my dog	
**Urgency to seek care?**	Please indicate how long you would wait to contact your veterinarian in the following circumstances:<br><br>If your dog became lame without having any visible injury or accident<br><br>If your dog began to scratch him or herself a lot without any obvious reason<br><br>If your dog began to soil the house after being housetrained for at least 1 year<br><br>If your dog had a soft lump on their neck that did not affect their behaviour<br><br>If you noticed your dog approaching the doors from the wrong side (where the hinge is), where before they seemed to know which way the doors open<br><br>If you thought your dog was overweight<br><br>If your dog lost their appetite<br><br>If your dog started acting aggressively or became fearful towards people<br><br>If you noticed your dog bumping into objects	5-point Likert scale:<br><br>Immediately seek an emergency appointment<br><br>On the same day to seek an appointment as soon as possible<br><br>Within a week, if the condition didn't improve<br><br>Within a month, if the condition didn't improve<br><br>I would not contact the vet for this
**Managing your dog's health in the future**	Please tell us about your future plans for management of your dog's health. You can consider how often you'd like to see the veterinarian or other healthcare	Open-ended

	specialists and for what reasons, and the impact of the COVID-19 pandemic.	
**About your vet**	I chose my vet because (select all that applies). If you attend more than one vet practice, please answer these questions thinking about your primary vet:	Someone recommended them to me<br>My friends/ family attend it<br>It's affordable<br>It's covered by my insurance<br>It's nearby<br>My vet takes time to explain things clearly to me<br>It offers specialist services that I wanted to use<br>My dog doesn't go to vet<br>Other
	How long have you been attending this veterinary practice?	Less than one year<br>1-3 years<br>More than 3 but less than 5 years<br>More than 5 but less than 7 years<br>More than 7 years.<br>Don't know/ can't remember
**About COVID-19 in your area**	Have you experienced suspected COVID-19 disease symptoms?	Yes

		No
	Has anyone else in your household experienced suspected COVID-19 disease symptoms?	Yes<br><br>No<br><br>No, not applicable- I live alone
	Are you classified as vulnerable and were you told to isolate for 12 weeks regardless of symptoms?	Yes<br><br>No<br><br>Not officially told but consider myself vulnerable
**About you**	What is your age?	18-29<br><br>30-49<br><br>50-59<br><br>60 or over<br><br>Prefer not to say<br><br>Recoding pattern: Under 50 years of age (18-29/30-49), Over 50 years of age (50-59/ 60 or over), Prefer not to say
	What is your gender?	Male<br><br>Female<br><br>Other (please specify)

		Prefer not to say
	What is your highest level of qualification?	University Higher Degree (e.g. PhD, MSc) *<br><br>First degree level qualification (including foundation degrees, graduate membership of a professional institute*<br><br>PGCE*<br><br>Diploma in higher education*<br><br>Teaching qualification (excluding PGCE) *<br><br>Nursing or other medical qualification not yet mentioned*<br><br>A-Level<br><br>Welsh Baccalaureate<br><br>International Baccalaureate<br><br>AS Level<br><br>Higher Grade/Advanced Higher (Scotland)<br><br>Certificate of Sixth Year Studies<br><br>GCSE/O-Level<br><br>CSE<br><br>Standard/Ordinary (O) Grade/Lower (Scotland)<br><br>Other school (including school leaving exam certificate or matriculation)

		None of the above (please describe)<br><br>Recoding pattern: above university level (variables marked with*), prefer not to say, below university level (all other variables)
	What's your household income?	Less than £15,000*<br><br>£15,000-20,000*<br><br>£20,001- £35,000*<br><br>£35,001-50,000<br><br>£50,001-65,000<br><br>£65,001- 80,000<br><br>£80,001- 95,000<br><br>£95, 001- 110,000<br><br>£110,001- 125,000<br><br>£125,001- 140,000<br><br>Over £140,000<br><br>Prefer not to say<br><br>Recoding pattern: Below or within national median (£29,900, O'Neil, 2021; variables marked with*), Above national median, Prefer not to say

	Are you worried about losing income due to the COVID-19 situation?	Yes, I'm very worried about losing income* <br>Yes, I'm somewhat worried about losing income* <br>I'm neither worried nor not worried about losing income§ <br>I'm generally not worried about losing income† <br>I'm not at all worried about losing income† <br>Don't know‡ <br>Prefer not to say‡ <br>Recoding pattern: Worried (variables marked with*), ambivalent (variables marked with§), not worries (variables marked with†), don't know or prefer not to say (variables marked with ‡)
	How would you describe your household composition?	I live alone <br>I live with others
	Current number of dogs:	None- my dog passed away/ was rehomed during the pandemic <br>1 <br>2 <br>3 or more
	How many dogs have you had before this one?	I had a dog/dogs growing up, but this is my first dog as an adult*

		This dog is my first dog ever* <br>I had 1 more dog as an adult§ <br>I had 2 more dogs as an adult§ <br>I had more than 2 dogs as an § <br>Recoding pattern: first dog during adulthood (variables marked with *), other dogs during adulthood (variables marked with §)
	Is there anything else you would like us to know?	Open-ended

[1]Potentially needing treatment was defined in the survey as "you were unsure whether it [veterinary care] was needed, but you considered it".

[2]Acute conditions were defined as "sudden-onset conditions, e.g. open wound, broken limb or tail, heatstroke, poisoning (e.g. eating chocolate/ plants), swallowed objects, teeth injury, eye/ear/skin/urinary infection (bladder/ kidney), torn nail or dewclaw, allergy, fits/ tremors, joint/ ligaments problems (e.g. cruciate ligament tear, hip dysplasia) or gastroenteritis (vomiting and diarrhoea)."

[3]In case where a dog needed help for more than one condition, respondents were asked to describe the earliest condition that a dog needed help for.

[4] Chronic conditions are defined as any health issues affecting a dog over a period of at least one year and likely to require ongoing medical attention, e.g. osteoarthritis (stiff/painful joints), epilepsy (symptoms may include seizures or funny spells), diabetes (changes in hormonal/endocrine system which lead to high blood sugar levels which may lead to changes in dog's appetite, thirst, changes in weight and needing to urinate more), dental problems (issues with teeth/ gums), skin problems (itching, allergies, skin sensitivity),ear disease or problems, or help with weight management.

Appendix B. Internal consistency (reliability) of HMB constructs and how these were used in logistic regression models.

Construct	Candidate variables	Cronbach's alpha	Outcome
**Perceived susceptibility**	'It's important to vaccinate dogs'	The third sentence showed poor	Perceived susceptibility- in models fitted as

	‘I think that providing my dog with regular veterinary check-ups or treatments is important’<br><br>‘My dog’s health deteriorated since the beginning of the pandemic’	consistency (alpha=0.23), so it was dropped. For the two remaining sentences, the<br><br>Raw alpha= 0.66, Standardised alpha= 0.67	both one variable (average of scores on the first two sentences) and individual sentences.<br><br>‘My dog’s health deteriorated since the beginning of the pandemic’ fitted as a stand-alone statement.
**Perceived severity (applicable only to dogs with chronic health issues)**	‘The treatment provided by my veterinarian is necessary to manage my dog’s health’ ‘Interrupting the treatment would be very risky’	Raw alpha= 0.72, Standardised alpha= 0.74	Perceived severity treated as one variable (average score for two questions)
**Perceived benefits**	‘The treatment and advice provided by veterinarian is necessary’<br><br>‘I trust my veterinarian’<br><br>‘My veterinarian is knowledgeable’, ‘I care about my vet’s views about how I manage my dog’s health’, ‘My vet thinks that providing my dog with regular check-ups or treatment is important’	‘I care about my friend’s and family’s views about how I manage my dog’s health’ had poor consistency (alpha= 0.52), so it was dropped.	The sentence ‘I care about my friend’s and family’s views about how I manage my dog’s health’ was fitted as a stand-alone sentence in the models. Perceived benefits treated as one variable

	‘My friends and family think that providing my dog with regular check-ups is important’,<br><br>‘I care about my friends and family’s views about how I manage my dog’s health’.	For the other variables, the raw alpha=0.81, standardised alpha=0.82	(averaged score for the 6 questions)
**Perceived barriers**	‘Managing my dog’s behaviour during veterinary consultations is/ has been difficult’<br><br>‘My past experiences with veterinarians were not always good’	Raw alpha= 0.26, Standardised alpha= 0.31	These variables were fitted as independent statements.
**Self-efficacy**	‘Managing my dog’s health is easy’<br><br>‘I feel well informed and knowledgeable about my dog’s health’<br><br>‘I know where to seek information about my dog’s health’<br><br>‘I am confident in recognising when my dog is not feeling well’<br><br>‘I am aware of different treatment options for my dog’s condition’<br><br>‘If I needed to, I would be able to access veterinary care for my dog’.	Raw alpha= 0.63, Standardised alpha= 0.66	Perceived self-efficacy- in models fitted first as one variable (average of scores on all sentences) and individual sentences.
